# Fecal microbiota transplantation treatment of autoimmune-mediated type 1 diabetes mellitus

**DOI:** 10.3389/fimmu.2022.930872

**Published:** 2022-08-12

**Authors:** Lina He, Rongping Chen, Bangzhou Zhang, Shuo Zhang, Barkat Ali Khan, Dan Zhu, Zezhen Wu, Chuanxing Xiao, Baolong Chen, Fengwu Chen, Kaijian Hou

**Affiliations:** ^1^ Department of Endocrine and Metabolic Diseases, Longhu People’s Hospital, Shantou, China; ^2^ Key Laboratory for Research on Active Ingredients in Natural Medicine of Jiangxi Province, Yichun University, Yichun, China; ^3^ School of Laboratory Medical and Biotechnology, Southern Medical University, Guangzhou, China; ^4^ School of Pharmacy, Fujian University of Traditional Chinese Medicine, Fuzhou, China; ^5^ School of Basic Medical Science, Central South University, Changsha, China; ^6^ Department of Endocrine and Metabolic Diseases, The First Affiliated Hospital of Shantou University Medical College, Shantou, China; ^7^ Drug Delivery and Cosmetics Lab, Good Clinical Practice (GCPS), Faculty of Pharmacy, Gomal University, Dera Ismail Khan, Pakistan; ^8^ Department of Gastroenterology, The Second Affiliated Hospital of Fujian University of Traditional Chinese Medicine, Fuzhou, China; ^9^ Center for Research and Development, Xiamen Treatgut Biotechnology Co. Ltd., Xiamen, China

**Keywords:** fecal microbiota transplantation (FMT), gut microbiota (GM), autoimmune-mediated, treatment, type 1 diabetes mellitus

## Abstract

Type 1 diabetes mellitus (T1DM) is an autoimmune-mediated disease characterized by a reduced or absolute lack of insulin secretion and often associated with a range of vascular and neurological complications for which there is a lack of effective treatment other than lifestyle interventions and pharmacological treatments such as insulin injections. Studies have shown that the gut microbiota is involved in mediating the onset and development of many fecal and extrafecal diseases, including autoimmune T1DM. In recent years, many cases of gut microbiota transplantation for diseases of the bowel and beyond have been reported worldwide, and this approach has been shown to be safe and effective. Here, we conducted an experimental treatment study in two adolescent patients diagnosed with autoimmune T1DM for one year. Patients received one to three rounds of normal fecal microbiota transplants (FMT) and were followed for up to 30 weeks. Clinical outcomes were measured, including biochemical indices, medication regimen, and dosage adjustment. Fecal microbiota metagenomic sequencing after transplantation provides a reference for more reasonable and effective microbiota transplantation protocols to treat autoimmune T1DM. Our results suggest that FMT is an effective treatment for autoimmune T1DM.

**Clinical Trial Registration:**

http://www.chictr.org.cn, identifier ChiCTR2100045789.

## Introduction

The development of T1DM is not only associated with genetic factors but also non-genetic factors, and rapidly changing external environmental factors especially play an important role in the pathogenesis of T1DM (1). The incidence of diabetes and impaired glucose tolerance in adults has been increasing worldwide, associated with changes in dietary habits and lifestyle, the use of antibiotics, changes in childbirth practices, and external environmental pollution (2–5). There is an estimated annual growth rate of 3%–4% of newly diagnosed diabetics (4, 6) and the International Diabetes Federation Atlas (IDF) estimates the global prevalence of diabetes to be 10.5% (536.6 million people) among people aged 20–79 years in 2021, rising to 12.2% (783.2 million) in 2045 (7), and 108,300 children under 15 years of age will be diagnosed in 2021, a number that rises to 149,500 if the age range is extended to under 20 years (8). T1DM is currently treated with lifestyle interventions, local insulin injections, and oral hypoglycemic agents. However, the main groups of T1DM sufferers are adolescents and children, and their blood glucose, which is often poorly controlled through diet and exercise, fluctuates. Additionally, long-term insulin use can cause a series of side effects such as obesity, hypoglycemia, hyperinsulinemia, and injection site pain. Importantly, it will also lead to unhealthy psychology in juvenile patients (9). Unhealthy psychology may also be involved in the developmental process of T1DM (10, 11). The diagnosis and management of diabetes also imposes a huge economic burden on the country and families (12). Therefore, novel treatment modalities for autoimmune type 1 diabetes have become an immediate social need, and gut microbiota transplantation is a global research hotspot.

Early in life, the gut microbiota shape the immune system and regulates metabolism, whereas imbalances in the gut microbiota later in life can cause several autoimmune and metabolic disorders (1, 8). Social and lifestyle changes, including the mode of delivery of newborns, dietary structure, and the use of antibiotics in the latter stages of life, have aggravated the imbalance in the type and quantity of fecal microbiota (13). These alterations have stimulated the exploration of the relationship between various internal and external fecal diseases and the change in the structure of fecal microbiota, the incidence of which is gradually increasing worldwide (2). The number of fecal microbiota, the types of dominant microbiota, and the proportion of microbiota were found to change in type 1 diabetic patients compared with normal people (9). Qi et al. (2) studied changes in the microbiota of children with T1D and healthy children (control group) and found that the fecal bacterial content was significantly lower in the T1D group than in the control group; it was also found that Clostridial microbiota IV and XIVa also play an important role in the homeostasis of the immune fecal environment and that their products, short-chain fatty acids, regulate the expression of Foxp3 in CD4+ T cells (3).

At least two mechanisms may explain the fecal microbiota involvement in the occurrence and development of T1DM. By one mechanism, fecal microbiota affect the permeability of fecal mucosa. Fecal microbiota secrete mucus to constitute the fecal mechanical barrier, and the breakdown products of fecal microbiota, short-chain fatty acids, promote the metabolism and repair of fecal epithelial cells and maintain the barrier function of fecal epithelium. Alternatively, fecal microbiota affect autoimmunity. Studies on NOD mice revealed that the interaction between fecal microbiota and the natural immune system is an important factor in the development of type 1 diabetes (4). When the fecal microbiota are dysregulated and the fecal barrier function is reduced, pathogenic microorganisms can invade the mesenteric lymph nodes, causing excessive activation of T and B lymphocytes in the intestine and intestine-associated lymphoid tissues. Since these tissues are connected to the pancreas through the pancreatic lymph nodes and mesenteric lymph nodes, the activated T and B lymphocytes trigger immune- and inflammation-mediated pancreatic β-cell injury (5), which further decreases insulin secretion.

The modulation of the gut microbiota through probiotics, prebiotics, interventions with dietary factors, and fecal microbial transplants all allows beneficial microorganisms to modulate early host–microbiota interactions by exerting their protective potential in patients with T1DM or those at high risk of T1DM (6). Among these therapeutic modifications, the main one is fecal microbiota transplantation (FMT). FMT is a method for regulating or rebuilding fecal microbiota and treating gastrofecal and non-gastrofecal diseases by fabricating a suspension of fecal microbiota from a healthy donor through an intelligent enterobacteria processing system and transplanting it into the gastrofecal tract of the patient by capsule preparation, nasal-fecal tube infusion, gastroscopy, enteroscopy, or intubation. The application of fecal microbiota transplantation has brought hope for the cure of many diseases, including irritable bowel syndrome (7), ulcerative colitis (10), and other fecal diseases, and depression, anxiety disorders (11), and many other diseases outside the fecal tract. Several examples of improved glycemic control in mice by gut microbiota transplantation have been described in rodent models (9–11). Transplantation of gut microbiota from MyD88-deficient non-obese diabetic (MyD88−/−NOD) mice into non-obese diabetic mice reduced the incidence of isletitis and significantly delayed the onset of diabetes (12). Administration of Lactococcus lactis expressing GAD65 and IL-10, combined with a short course of low-dose anti-CD3, stabilized isletitis, preserved functional beta-cell populations, and restored blood glucose to normal levels in recently developed NOD mice (14). Additionally, Dolpady et al. (11) found experimentally that alterations in the fecal microbiota induced by oral administration of the lactic acid bacteria-rich probiotic VSL#3, alone or along with retinoic acid (RA), protected NOD mice from T1D by affecting the inflammasome at the fecal level. Thus, FMT can adjust the fecal microbiota of transplanted patients and slow down T1MD disease progression.

FMT was found to be safe and effective in several human clinical studies. FMT has significantly altered the composition of the gut microbiota and to affect glycemic control and insulin resistance in subjects with metabolic syndrome based on baseline microbiota (15); therefore, in recent years, greater research attention has been focused on fecal microbiota transplantation for T1DM. Mocanu et al. (16), studied the change in insulin sensitivity (Homa-IR) from baseline to 6 weeks after fecal microbiota transplantation supplemented with high and low fermentable fiber in patients with obesity and metabolic syndrome, and found that FMT combined with low fermentable fiber significantly improved insulin resistance in transplant recipients. The difference was statistically significant, with no cases of adverse effects throughout the transplantation process. In performing lean body donor (allogeneic) versus self (autologous) fecal microbiota transplantation in male subjects with metabolic syndrome, Kootte (17) found a significant improvement in insulin sensitivity after 6 weeks of allogenic fecal microbiota transplantation, accompanied by changes in microbiota composition and changes in plasma metabolites, with no cases of adverse effects during this experiment. In a randomized controlled trial of patients with new-onset T1DM within 6 months, de Groot and colleagues (16) found that fecal microbiota transplantation stabilized residual beta-cell function and optimized glycemic control in patients. These clinical trials show that fecal microbiota have a great biological driving effect in T1DM patients, and FMT is a safe and feasible treatment of T1DM. Microbiota transplantation has also been reported in obese people with type 2 diabetes (18).

To understand clinical strategies to treat T1DM, we initiated an experimental treatment study in two adolescent pediatric patients with autoimmune T1DM. Ianiro et al. (19) conducted a meta-analysis that showed that the overall response rate of capsule FMT was over 90% and was minimally invasive, and Ng et al. (18) found that repeat FMT is safe and facilitates the implantation of the transplanted microbiota. Therefore, we chose to perform multiple clinical nodes of fecal microbiota transplantation, most as oral liquid capsules, in our patients. The number of performed microbiota transplants and the specific clinical pathways differed between the two patients, resulting in different clinical outcomes, as will be discussed.

## Materials and methods

At the onset, we took stool specimens from the subjects and mated them with healthy donors who met the criteria. Both patients had signed informed consent for microbiota capsule preparation when they were enrolled. The mated fecal microbiota was introduced into the patients in oral capsules or by nasal-fecal tube infusion, and serum and stool specimens were collected for observation and laboratory analysis before and after each microbiota transplantation was completed. Current research on diabetes has focused on the collection and analysis of serum samples from patients, but it is also important to collect stool samples and correlate a multi-omics approach with the gut microbiota because only then can we reconstruct the bridge from disease parameters to etiology (6]. Therefore, our observational indices are divided into two types: 1) primary observation indices, i.e., changes in insulin and oral hypoglycemic drug use compared with those before microbiota transplantation, glycemic control, insulin resistance, and whether autoantibodies associated with autoimmune T1DM turn negative; and 2) secondary observation indices, by metagenomic analysis of the fecal microecological changes of the patients, fluctuations in inflammatory factors (blood routine, white blood cells, sedimentation, C-reactive protein, calcitoninogen), and linking the serological changes and fecal microbiota changes of the patients to each other. The study procedure is described below.

Note: Research ethics number: LHLL2021001, Ethics Committee of Longhu People’s Hospital Shantou, China.

Clinical Registration Number: ChiCTR2100045789, Chinese Clinical Trail Registry: http://www.chictr.org.cn/showprojen.aspx?proj=125179.

### Recruitment criteria for study subjects

Recruitment parameters for patients followed the 2019 World Health Organization (WHO) diagnostic criteria for type 1 glucose (T1DM). The inclusion and exclusion criteria are shown in **Table 1**.

**Table 1:** Inclusion and Exclusion criteria for subjects.

Inclusion criteria	Exclusion criteria
1 Have detectable production of autoantibodies that cause impaired islet cell function (ICA or/and IAA or/and GAD antibody positive); 2. after stable glycemic control with intensive insulin therapy, islet function C peptide release levels assessed under stimulation of a mixed diet remain below normal or lower limit of detection; 3. any gender; 4.any age (patients older than 18 years old should voluntarily participate in the trial and have good compliance; 5. younger than 18 years old should obtain informed consent from one and, if necessary, both guardians and have good compliance); 6. any body mass index; and 7. must be able to cooperate with the follow-up of the study team.	1. combination of acute and chronic infectious diseases, gastrofecal diseases, severe cardiac insufficiency, severe hepatic and renal insufficiency, leukopenia or leukopenia, autoimmune disease manifestations or previous diagnosis of autoimmune disease in the past three months, other diseases or complications; 2. other gastrofecal diseases that may affect drug absorption; 3. pregnant and lactating women; 4. patients treated with other hormones or antibiotics in the past three months; 5. new onset of cardiovascular disease within the last three months; and 6. participated in other clinical trials during the same period.

Based on the above recruitment criteria, two patients, designated patient 1 (P1) and patient 2 (P2), were included in this study.

Patient 1 was a 15-year-old male, with a height of 1.55 m, a weight of 37 kg, and a body mass index (BMI) of 15.4 kg/m2, who had been diagnosed with T1DM for more than 1 year. Prior to FMT, the patient required insulin in combination with oral hypoglycemic agents for glycemic control. Under pharmacological treatment, FBG and 2HPG were moderately controlled with a small fluctuation range. The first FMT for this patient was performed by transnasal fecal tube injection of 200 ml of precisely matched bacteriological solution provided by the South China Bacterial Bank, while the second and third FMTs were subsequently performed at the sixth and eighth week after the first transplantation by oral administration of bacteriological solution capsules. Several serum and fecal samples were collected, and a 30-week clinical follow-up was performed.

Patient 2 was a 12-year-old male patient diagnosed with T1DM for 1 year. He was 1.51 m tall, weighed 31 kg, and had a BMI of 13.8 kg/m2. We performed two fecal microbiota transplants in the form of oral liquid capsules on 29 July 29 and 2 August 2021, during which multiple serum and stool samples were collected from the patient and a 10-week clinical follow-up was performed. Prior to the microbiota transplantation, the blood glucose of the patient was controlled with insulin and oral hypoglycemic agents, with FBG fluctuating from 7 to 8 mmol/L and 2 HPG fluctuating from 12 to 21 mmol/L. The usual self-life management of the patient was poor, with a wide range of blood glucose fluctuations.

### Fecal donor recruitment, randomization, and FMT procedures

Healthy individuals after a comprehensive screening (20) were accepted as donors for fecal microbiota transplantation, and patients meeting the inclusion criteria were designated as recipients. Subjects received the same diet and exercise controls. The dietary principles of both diabetic patients were low salt, low fat, and light during the transplantation period. The two patients followed the guidelines of waiting for 1 h to exercise after meals, with an exercise time of 30 min a day and regular exercise and daily activities for five days a week. Diabetes-related indicators and inflammatory factors were recorded and evaluated before starting treatment (see **Table 1**). In a study of FMT for intestinal inflammatory diseases (10, 21, 22), it was found that FMT at weeks 1, 6, and 8 could significantly improve the composition of intestinal microbiota, endoscopic disease severity index, and inflammatory index, and frequent intensive regimens were effective (23, 24). Therefore, we chose to give patients three rounds of transplantation at the time points of day 1, week 6, and week 8. Among the two patients included in our study, one patient completed the transplantation at all-time points, and the other patient only completed one round of flora transplantation because of the problem of compatibility. Patient 1 received three fecal microbiota transplants on day 1, week 6, and week 8 of the experiment by one nasal fecal tube injection and two oral capsules, respectively. Patient 2 received two fecal microbiota transplants by oral capsules on days 1 and 4 of the experiment. Both patients were evaluated by collecting stool specimens after each microbiota transplant.

### Clinical indicators and relevant laboratory methods applied

Blood (5 ml) was drawn from the elbow vein of the subject before treatment and at the corresponding clinical node after each fecal microbiota transplant treatment in the early morning on an empty stomach, and the samples were collected and centrifuged at 3,000 rpm, and the serum was separated. Fasting glucose (FBG), 2-hour postprandial glucose (2HPG), 2-hour postprandial insulin (2INS), fasting insulin (FINS), fasting C-peptide (FCP), 2-hour postprandial C-peptide (2HCP), white blood cell (WBC), blood sedimentation (ESR), C-reactive protein (CRP), calcitoninogen (PCT), biochemical index, and steady-state model, which included insulin resistance index (Homa IR) and islet cell function index (HOMA-HBCI), where Homa IR = (FINS × FPG)/22.5 and HOMA-HBCI = 20 × FINS/(FPG − 3.5)), glycated hemoglobin was measured by ELISA. Data are expressed as mean values ± standard deviation. Unpaired Student’s t-test for categorical variables was applied for comparison between before and after FMT. Results with a two-tailed p-value of <0.05 were considered significant. IBM SPSS Statistics 25 was used for clinical index analyses.

Simultaneously, stool specimens were collected at different time points after each microbiota transplantation treatment. Then the stool specimens were stored in a microbiota stabilizer, EffcGut (25) until DNA extraction. Fecal genomic DNA was extracted using the QIAamp Fast DNA Stool Mini Kit (Qiagen, CA, USA). DNA samples were fragmented to an insert size of 400 bp for library preparation and sequenced by Illumina Nova seq with PE 150 reagents. Raw reads were detected as having good sequencing quality by FastQC (http://www.bioinformatics.bbsrc.ac.uk/projects/fastqc/) with default parameters. Then reads were trimmed to filter the sequencing adapter, low-quality reads, and the human genome (based on reference hg18) using Trimmomatic and Bowtie2 with default parameters (26, 27).The microbial taxonomic composition at genus, species, and strain levels was processed using default parameters (25). The metagenome sequencing was used to: 1) compare and analyze the alpha diversity (including the abundance (ACE, Chao1, and observed species), diversity (Shannon index and Simpson index), homogeneity (J), and beta diversity of the fecal microbiota of each patient before and after transplantation, the latter transplantation compared with the previous transplantation, and the later long clinical follow-up; 2) compare and analyze the microbiota with the same trend at the species and genus levels for both patients after fecal microbiota transplantation; and 3) make an analysis of blood glucose related indexes and fecal microbiota correlation at the species and genus levels. These analyses were performed using R v3.4.1 (28) and the main R packages were ggplot2 (v3.3.5) (29) and vegan (2.5–7) (30).

## Results

### Adjustment of drug regimen and changes in serum indices before and after transplantation in two patients

**Figure 1:**
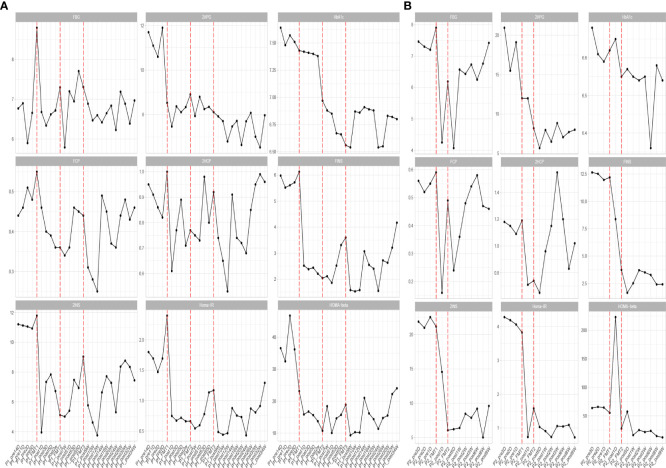
Changes in blood glucose before and after transplantation in Patients 1 **(A)** and 2 **(B)**. FBG, fasting blood glucose; 2HPG, 2-hour postprandial blood glucose; HbA1c, glycated hemoglobin; FCP, fasting C-peptide; 2HCP, 2-hour postprandial C-peptide; FINS, fasting insulin; 2INS, 2-hour postprandial insulin; Homa-IR, insulin resistance index; HOMA, islet cell function index. The horizontal coordinates in the figure indicate the different time points before and after transplantation. The vertical coordinates are the corresponding values of glucose-related clinical indicators at different time points.

Patient 1: During the transplantation process and the later clinical follow-up, our observation indicators were annotated as follows:

Adjustment of medication regimen. Within one week after the completion of the first FMT, the patient had stopped taking insulin as well as oral hypoglycemic drugs, and no additional hypoglycemic drugs were added during the subsequent FMT and clinical follow-up. Until the end of the follow-up period, the FBG and 2HPG of the patient did not fluctuate significantly and were not significantly different from those under insulin control before transplantation, so FMT brought beneficial changes to the patient (**Figure 1**).

Blood glucose-related indices. As shown in **Figure 1**, the FBG and 2HPG of the patient had a large decrease after the first FMT and after each subsequent FMT. After each FMT, FBG and 2HPG decreased, though the decrease was smaller than the first one. Three days after each transplant, the trend of blood glucose fluctuation gradually stabilized, and based on the discontinuation of hypoglycemic drugs, the overall blood glucose of the patient showed a trend of first decrease and then stable change. As shown in **Table 2** (Patient 1 blood glucose index), the FBG of the patient after treatment (6.77 ± 0.44 mmol/L) was lower than that before treatment (7.01 ± 1.08 mmol/L), p = 0.056, though this was not statistically significant. However, the 2HPG of the patient was significantly lower after treatment (7.82 ± 0.65 mmol/L after compared to 10.77 ± 1.35 mmol/L before), p = 0.076, though this was not statistically significant. Given that the patient discontinued all hypoglycemic agents, including insulin, after the fecal microbiota transplants, FMT was beneficial to the patient. The glycosylated hemoglobin of the patient decreased to a certain extent after each transplantation, and after about 2 weeks after the completion of all transplants, the glycosylated hemoglobin increased slightly and then stabilized, but the overall level still showed a decreasing trend (**Table 2**). The mean glycated hemoglobin was significantly better controlled in the patient after transplantation (**Table 2**). As seen in **Figure 1**, the C-peptide and insulin secretion levels of the patients showed a decreasing trend after each transplantation, while C-peptide and insulin levels showed an increasing trend about 3 months after the completion of transplantation. Homa-β had a substantial decrease after the first transplantation, which was mainly caused by the discontinuation of exogenous insulin. In the later clinical follow-up, there was a small increase in Homa-β, indicating an upward trend in the own islet secretion function of the patient. Homa-IR decreased substantially to normal (<1) after the first transplantation, and then fluctuated within a small range, but at the follow-up of the 30th week, Homa-IR had increased again to >1. The C-peptide, insulin secretion index, and Homa-IR in the patient were reduced after transplantation compared with those before transplantation (**Table 2**). This was mainly due to the presence of a large amount of exogenous insulin in the body of the patient before FMT interfered with the before-and-after comparison of these clinical indicators. There is no washout period in this study because insulin withdrawal in type 1 diabetes will lead to a significant increase in blood glucose and may lead to diabetic ketosis or even diabetic ketoacidosis. The patient was observed after FMT and discontinuation of exogenous insulin treatment. The Homa-IR, the index of insulin resistance, was calculated. We found that the value of Homa-IR fluctuated within the normal range (0.75 ± 0.24, <1) after transplantation, and that the insulin resistance of this patient improved obviously, though this was not statistically significant.

**Table 2:** Mean values of blood glucose and insulin indexes plus or minus standard deviation at each time point before and after treatment of Patients 1 and 2.

Group	FBG	2HPG	HbA1c	FCP	2HCP	FINS	2INS	Homa-IR	Homa-β
	mmol/L	mmol/L	(%)	ng/ml	ng/ml	mU/L	mU/L		
Patient1 Before fmt	7.01 ± 1.08	10.77 ± 1.35	7.53 ± 0.08	0.49 ± 0.04	0.91 ± 0.07	5.79 ± 0.26	11.22 ± 0.33	1.81 ± 0.35	35.03 ± 8.5
Patient1 After fmt	6.77 ± 0.44	7.82 ± 0.65	6.89 ± 0.29	0.40 ± 0.07	0.79 ± 0.13	2.48 ± 0.71	6.62 ± 1.58	0.75 ± 0.24	15.22 ± 4.11
P value	0.056	0.076	0.126	0.173	0.118	0.137	0.006	0.49	0.115
Patient2 Before fmt	7.47 ± 0.31	16.89 ± 3.96	6.63 ± 0.04	0.56 ± 0.03	1.15 ± 0.04	12.33 ± 0.31	21.64 ± 0.71	4.09 ± 0.2	62.45 ± 4.77
Patient2 After fmt	6.08 ± 1.08	7.90 ± 1.71	6.55 ± 0.07	0.43 ± 0.13	0.99 ± 0.27	3.39 ± 1.88	8.27 ± 2.70	0.97 ± 0.26	44.02 ± 64.24
P-value	0.16	0.026	0.72	0.052	0.047	0.246	0.184	0.619	0.195

FBG, Fasting glucose; 2HPG, 2-hour postprandial glucose; HbA1c, glycated hemoglobin; FCP, fasting C-peptide; 2HCP, 2-hour postprandial C-peptide; FINS, fasting insulin; 2INS, 2-hour postprandial insulin; pre-treatment and post-treatment values were given as mean plus or minus standard deviation; statistical analysis was performed using independent sample t-test with a significance level of p <0.05.

**Figure 2:**
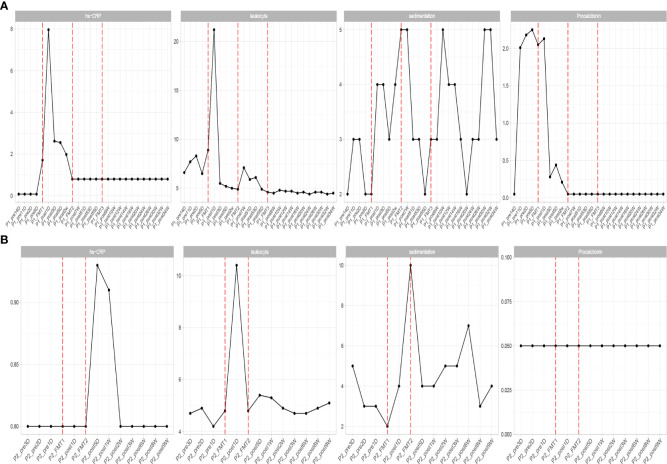
Changes in inflammatory indexes in patients before and after transplantation in Patients 1 **(A)** and 2 **(B)**. hs-CRP, C-reactive protein; leukocyte, white blood cells; sedimentation, blood sedimentation; Procalcitonin, calcitonin.

**Figure 3:**
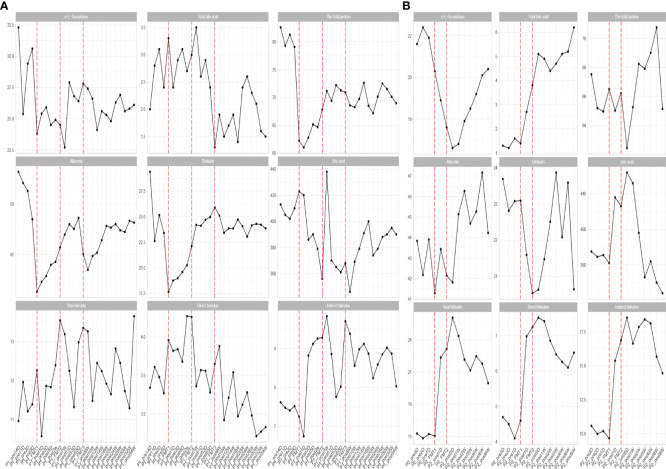
Changes in biochemical parameters before and after transplantation in Patients 1 **(A)** and 2 **(B)**. The horizontal coordinates in the graph indicate the different time points before and after the transplantation of the patient. The verticalcoordinates are the corresponding values of biochemical related indexes at different time points.

Inflammatory indexes. Before FMT, the inflammatory indices such as CRP, WBC, and ESR of the patient were within the normal range, while calcitonin indices were outside the normal range (**Figure 2**). Some studies have found that inflammatory responses unrelated to infection exist in type 1 diabetic patients, which may be due to an autoimmune disorder (15). After the initial transplantation, the leukocytes in the patient showed an acute increase to 21.2 × 109/L on the first day after transplantation (**Table 2**), and then decreased to normal after 2 days, while the rest of the inflammatory indices did not change. We considered that the rise of leukocyte indices might be related to the immune response triggered by the initial exposure to the transplanted microbiota in the body of the patient. Likely, the immune system of the patient had already adapted to the implantation of foreign microbiota, and this finding was beneficial for the progress of microbiota transplantation treatment. In the biochemical indices of the patient, these values always fluctuated within the normal range (**Figure 3**). The biochemical indices of the patient were normal before and after the treatment. These findings suggest that fecal microbiota transplants will not adversely affect the liver and kidneys of the patient.

Patient 2: Our observation indices were as follows:

Adjustment of medication. After the first FMT, the patient stopped the use of insulin and only took the oral hypoglycemic drug Repaglinide ligliptin. In the later clinical follow-up, the patient did not use insulin again, and the use of hypoglycemic drugs was as before.

Glycemic indices. The FBG of the patient fluctuated from 8 to 8 mmol/L and 2HPG fluctuated from 12 to 21 mmol/L (**Figure 1**). The FBG of the patient decreased after each transplant, with a larger decrease after the first transplant, and it rose again 1–2 weeks after completing the transplant, and then maintained at the impaired fasting glucose level (6–7 mmol/L) fluctuation, compared with the better improvement of the 2HPG of the patient (**Figure 1**). In contrast, the 2HPG of this patient improved, with some decrease in 2HPG after each transplantation and small fluctuations in 2HPG within the normal range after completion of transplantation and during the later clinical follow-up. As shown in **Table 2**, the post-treatment fasting glucose value (6.08 ± 1.08 mmol/L) of the patient was significantly lower than the pre-treatment fasting glucose value (7.47 ± 0.31 mmol/L) (p = 0.16). The post-treatment 2-hour postprandial glucose (7.90 ± 1.71 mmol/L) of the patient was also significantly lower than the pre-treatment 2-hour postprandial glucose (16.89 ± 3.96 mmol/L). As shown in **Figure 1**, the glycosylated hemoglobin of the patient decreased slightly after each transplantation and remained stable after the completion of transplantation. The glycosylated hemoglobin level after fecal microbiota transplants (6.55 ± 0.07%) of the patient was not significantly different from before fecal microbiota transplants (6.63 ± 0.04%). The C-peptide of the patient decreased after both transplants, and after a large increase, FCP remained basically stable, while 2HCP fluctuated in a wide range. The insulin index of the patient had a large decrease after the first fecal microbiota transplant. The change was related to the discontinuation of insulin, and then fluctuated at a lower level. In the later clinical follow-up, FINS and 2IS had a small upward trend. Homa-β decreased significantly after the first microbiota transplantation and remained stable afterwards. Homa-IR also decreased after the first microbiota transplantation and remained normal afterwards (0.97 ± 0.26), suggesting that the insulin resistance of this patient was greatly improved (**Table 2**).

Inflammatory indices: the inflammatory indices of the patient, such as C-reactive protein, blood leukocytes, and sedimentation, were within normal limits (**Figure 2**). As in the previous experimental cases, on the first day after the first transplantation, the patient showed an abnormal increase in leukocytes that was not accompanied by an increase in other inflammatory indices, and after 2 days the leukocyte index decreased to normal, and after the second fecal microbiota transplantation there was no abnormal increase in leukocytes. This phenomenon is consistent with our previous conjecture that the initial input of transplanted microbiota induces a transient inflammatory reaction in the patient, who will adapt to the transplanted microbiota and the inflammatory index will gradually decrease to normal. The subsequent transplantation of microbiota did increase the inflammatory index of the patient.

Biochemical indices: The biochemical indices of the patient before and after treatment were both normal (**Figure 3**), which suggested that the microbiota transplantation did not have adverse effects on the liver, kidneys, and other organs of the patient. In addition to this, both patients had no adverse effects after FMT.

The clinical outcomes of the two patients after transplantation showed that both patients had fair glycemic control during transplantation and later clinical follow-up based on discontinuation of all hypoglycemic agents and discontinuation of insulin, respectively, which indicates the effectiveness of FMT on glycemic control in T1DM and that this effectiveness can be maintained, which is consistent with the study by Mokhtari et al. (18); patients with T1DM who underwent fecal microbiota transplants effectively prolonged the function of residual beta cells. FMT significantly improved insulin resistance in both patients, which provides strong evidence for us to conduct more fecal microbiota transplants for T1DM in the future. The postprandial glucose fluctuation range of Patient 1 was smaller, and the improvement of insulin resistance was more obvious, which may be caused by the following reasons: 1) Patient 1 had more transplants than Patient 2, and the interval was longer, so the multiple transplants with a slightly longer interval may have a “strengthening” effect in improving blood glucose; 2) Patient 1 usually had better personal dietary management than Patient 2.

### Analysis of gut microbiota in two patients after transplantation

**Figure 4:**
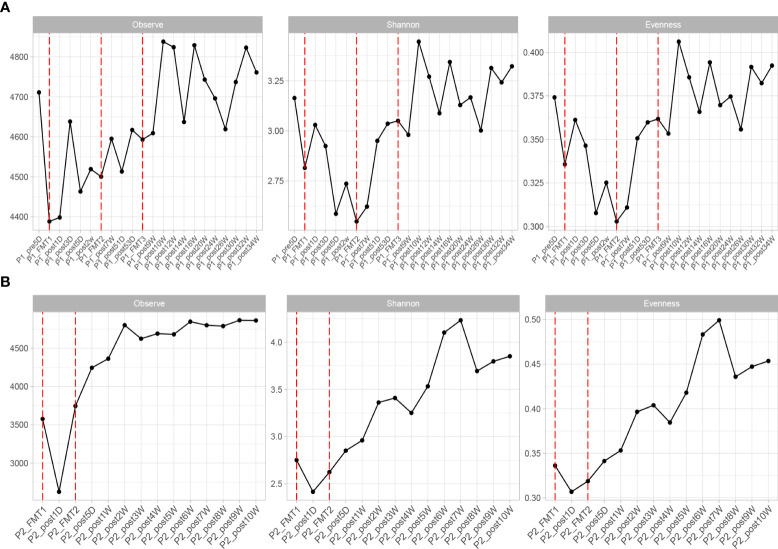
Changes in microbial diversity after transplantation in Patient 1 **(A)** and 2 **(B)**, including: Observe, Shannon, and Evenness index.

The altered microbial diversity of patients 1 and 2 is shown in **Figure 4**. Then, alpha-diversity analysis was used to analyze the complexity of microbial community composition within the samples, including the richness (ACE, Chao1, and observed species), diversity (Shannon and Simpson), and evenness (J) of the microbial community within the sample. The ACE, Chao1, observed species, Shannon and Simpson, and J indices of the fecal microbiota of Patient 2 showed an increasing trend after both transplants and stabilized at 16 days after transplantation, as shown in **Figure 4**.

**Figure 5:**
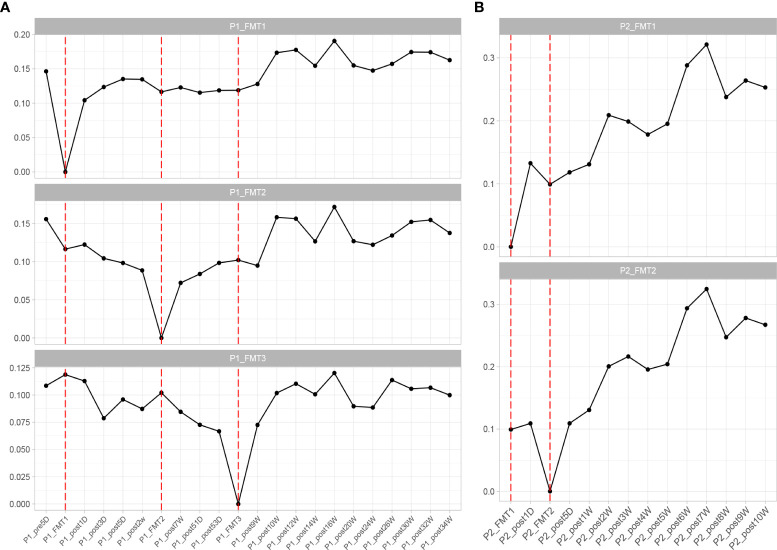
Changes in overall microbial composition of Patient 1 **(A)** and Patient 2 **(B)** after each transplant, measured by Bray–Curtis distance.

The change in microbial diversity in patient 1 and patient 2 is shown in **Figure 4**. The ACE, Chao1, and observed species indices showed increasing and stabilizing trends. The Shannon and Simpson and J indices fluctuated after the first transplant, but also showed an increasing and stabilizing trend after the second and third transplant. The overall microbial structure changed after FMT and tended to stabilize over time (**Figure 5** and **Figure S1**). Diversity improved with the first, second, and third transplants.

**Figure 6:**
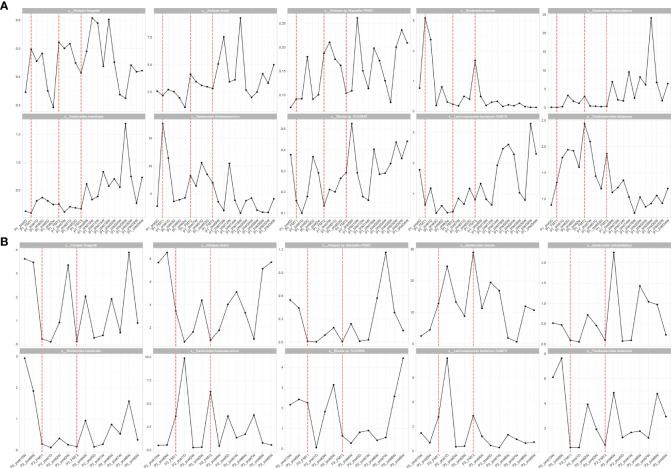
Patients 1 **(A)** and 2 **(B)** transplanted with consistent changes in microbial species level.

**Figure 7:**
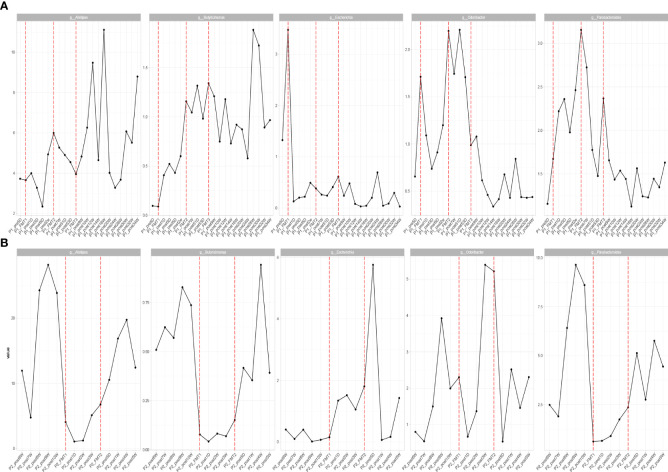
Patients with consistent changes in genus level after transplantation in Patient 1 **(A)** and Patient 2 **(B)**.

By high-throughput sequencing, we identified microbes that changed consistently after the transplantation of Patients 1 and 2. Both species level and genus level were included. At the species level (**Figure 6**), the bacteria that increased in both patients after transplantation were Alistipes sp. Marseille-P5997, Bacteroides cellulosilyticus, A. finegoldii, A. shahii, B. caccae, B. thetaiotaomicron, Blautia sp. SC05B48, and Lachnospiraceae bacterium GAM79. The genera (**Figure 7**) that both increased after transplantation were Butyricimonas, Alistipes, and Parabacteroides, and those that both decreased were Escherichia and Odoribacter.

Additionally, we performed correlation analysis between characteristic bacteria and clinical indicators at the genus level and species level. The correlation analysis of blood glucose related indicators and fecal microbiota at the genus level is shown in **Figure 8-1**. It can be found that the genera with a significant negative correlation with HOMA-beta are Faecalibacterium and Butyricimonas. Homa-IR was significantly negatively correlated with Butyricimonas and Phascolarctobacterium. 2HCP is significantly positively correlated with Streptococcus and Blautia. 2HCP was significantly negatively correlated with Phascolarctobacterium and Paraprevotella. Fasting C-peptide and Streptococcus were significantly positively correlated.

**Figure 8-1:**
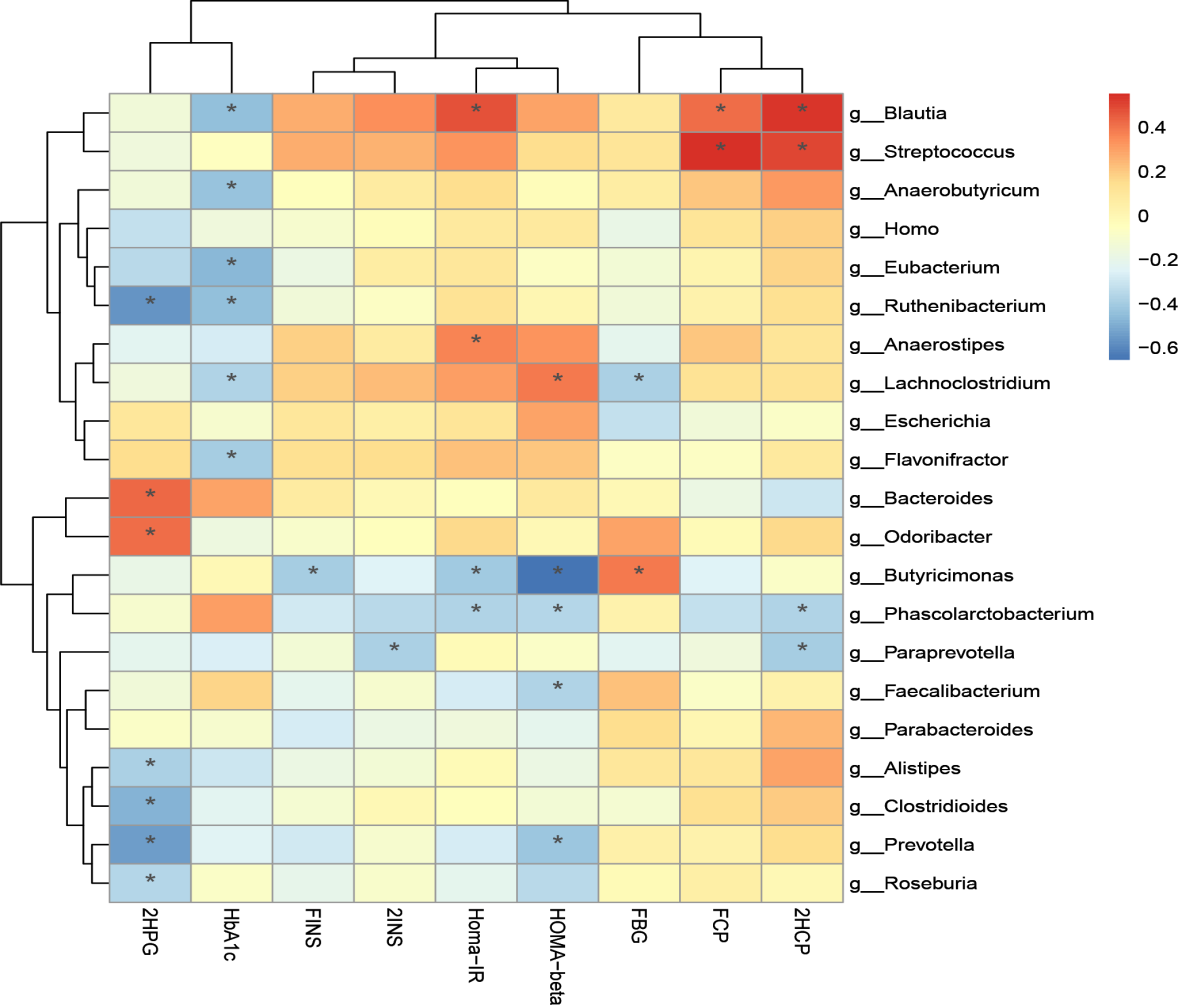
Correlation analysis of clinical indicators and genus level difference bacteria after transplantation in Patients 1 and 2 *P-value <0.05.

The correlation analysis of blood glucose related indexes and fecal microbiota at the species level is shown in **Figure 8-2**, and it can be found that HOMA-beta and L. bacterium GAM79 and B. caeccae were positively correlated, while B. salanitronis and F. prausnitzii were negatively correlated. The bacteria that showed a significant positive correlation with Homa-IR were B. ovatus. L. bacterium GAM79 and 2INS showed a significant positive correlation. L. bacterium GAM79, Ruminococcus. gnavus, L. bacterium Choco8, A. shahii, and B. ovatus were significantly positively correlated with 2HCP. P. succinatutens, P. faecium, and 2HCP were significantly negatively correlated. L. bacterium GAM79, Clostridium boleae, and B. caccae were significantly and negatively correlated with FBG. The correlation analysis between other clinical indicators and fecal microbiota was positive for A. finegoldii and ALP, total bile acid, and cystatin.

**Figure 8-2:**
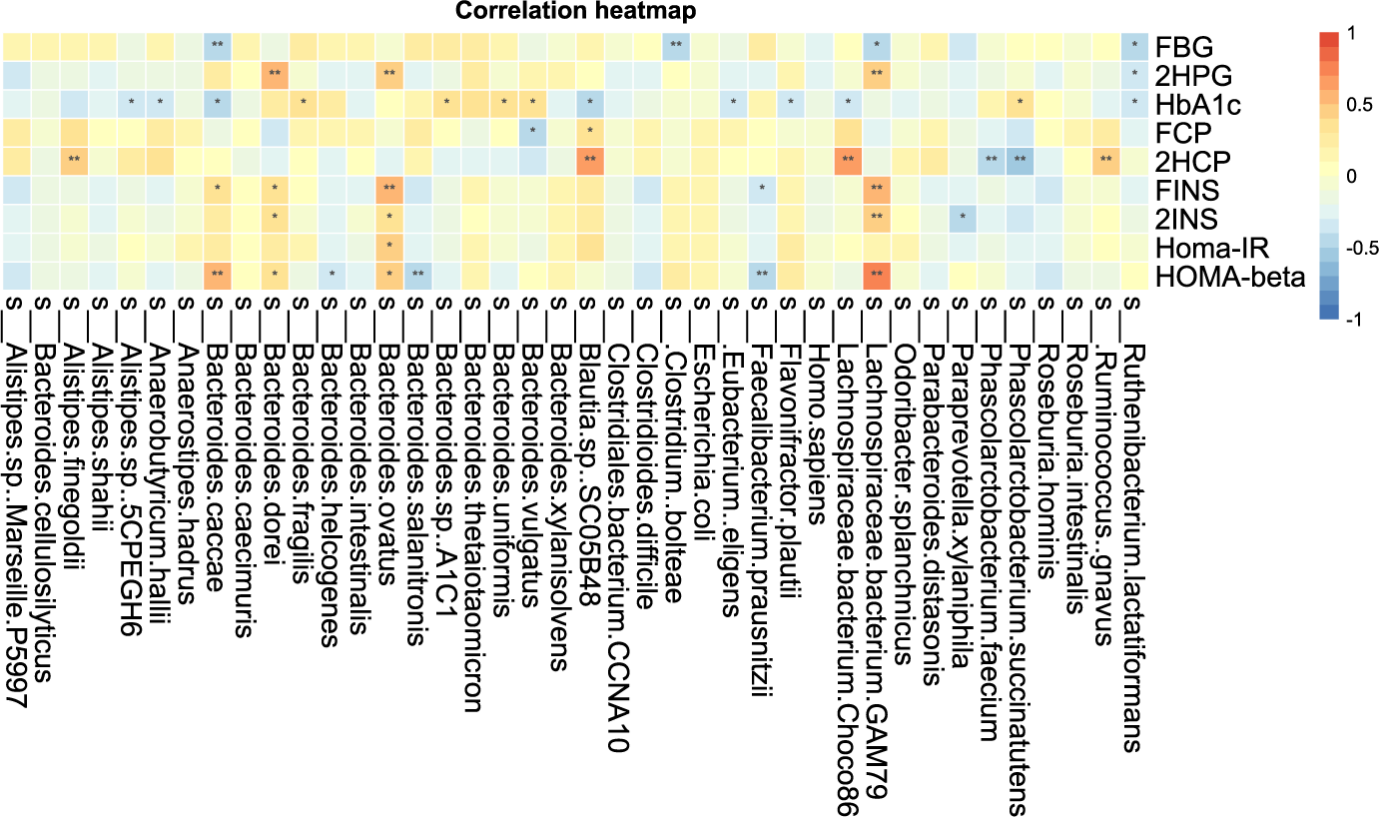
Correlation analysis of clinical indicators and species level difference bacteria after transplantation in Patients 1 and 2 *P-value <0.05, **P-value <0.01.

The correlation analysis of clinical indicators and microbiota at genus and species levels indicates that improvement of clinical indicators is associated with altered microbiota diversity.

## Discussion

T1DM is a metabolic disease characterized by insulin deficiency and disorders of glucose metabolism caused by autoimmune-mediated selective islet beta cell damage (34). The association between gut microbiota and autoimmune diseases is well known. Disruption of the microbiome microbiota balance can induce autoimmune diseases in people with certain genetic backgrounds and environmental factors (31). Many studies have found that dysbiosis is also involved in the pathogenesis of T1DM. In a Chinese study, children with T1 diabetes had a lower abundance of microbiota in stool samples compared to healthy controls, particularly the newly isolated gram-positive and butyric acid-producing anaerobic bacterium Inestinimonas (3). In contrast, increased glaucoma was found in these patients. In addition, early supplementation with probiotics has been shown to reduce the risk of islet autoimmunity in children at higher genetic risk for T1DM in a multicenter prospective cohort study (32). This means that the pathogenesis and development of T1D is driven by a combination of genetic susceptibility and environmental factors. The gut microbiota is one of the potential environmental influences involved in the pathophysiological processes of T1DM. Gut microbes mediate the development of diabetes by altering fecal permeability and modulating fecal immunity and molecular mimicry. Compared to healthy individuals, T1DM patients have significantly altered gut microbial diversity, taxonomic profile, and functional potential (33). In this study, two patients with T1DM received FMT, and after transplantation, both patients showed significant improvement in diabetes-related clinical indicators. Moreover, the diversity and abundance of fecal microbiota increased relative to pre-transplantation. Our study also found a correlation between the improvement of some clinical indicators of diabetes and the change of fecal microbiota and identified the genus and species of bacteria that increased and decreased in both patients after transplantation. Identification of the role of related bacteria in the development and progression of T1DM can help guide early screening and diagnosis of T1DM and the development of new target therapies for T1DM based on microecological reconstruction.

The bacteria that were reduced at the species level in both patients after transplantation were A. caccae, B. thetaiotaomicron, Blautia sp. SC05B48, and L. bacterium GAM79. A. caccae is a ubiquitous anaerobic bacterium. Patients with diabetes are at an increased risk of anaerobic infections. It has been shown that L. bacterium is a fecal conditionally pathogenic bacterium. It can invade the fecal mucosa and cause various abdominal septic infections (35). This study shows that the abundance of A. caccae can be reduced by microbiota transplantation, which also reduces the risk of A. caccae infection in diabetic patients. B. thetaiotaomicron has two key aspects in the human fecal symbiotic lifestyle: extensive digestion of dietary fiber and host polysaccharides, and dynamic cell surface structures that promote interaction and evasion with the host immune system. Like other fermentative gut microbes, polymorphic bacilli produce host-absorbable short chains and organic acids, both of which can be absorbed by the host as energy sources. The L. bacterium GAM79 decreased after transplantation in the two patients. The presence of lipopolysaccharide (LPS) in the intestine is necessary but insufficient for developing diabetes. The investigators hypothesized that Trichophyton spp. AJ110941 may help translocate LPS from the intestine to the bloodstream. It has been proposed that colonization by AJ110941 may promote pancreatic β-cell dysfunction. Trichoderma spp. AJ110941 may be one of the important pathogenic bacteria of T2DM (36). Our study concluded that the abundance of L. bacterium GAM79 could be reduced by fecal microbiota transplants, which may be beneficial for diabetic patients.

The genera with increased levels in both patients after transplantation were Vibrio butyricus, Vibrio aliens, and Vibrio parapsilosis. V. butyric acid generates butyric acid by fermenting glucose, which in turn synthesizes short-chain fatty acids that protect the structural and functional stability of the fecal epithelium and act to inhibit inflammation. The presence of V. butyricus spp. enhances fecal immunity, broad-spectrum antibacterial, and inhibits inflammatory responses. Children with T1DM have dysbiosis of the fecal microbiota and small numbers of P. aeruginosa and V. butyricus spp (37). In the case report of this study, both type 1 diabetic patients had an increase in V. butyricus spp. after microbiota transplantation, which is beneficial for type 1 diabetic patients. Both patients also had an increase in V. paraquaternus spp. after transplantation. Previously, the metabolic benefit of Dictyostelium paraquaternum was observed in reducing hyperglycemia in T1DM rats (38, 39). Treatment with Dictyostelium parapsilosis significantly altered the bile acid profile with elevated lithodeoxycholic acid and ursodeoxycholic acid and increased fecal succinic acid levels. In vitro cultures of D. parapsilosis have demonstrated its ability to convert bile acids and produce succinate. Supplementation with succinate in the diet reduced hyperglycemia in obese mice by activating fecal gluconeogenesis. This means that the bacterium reduces obesity and metabolic disorders by producing succinate and secondary bile acids (40). The genera in which the levels in both patients decreased after transplantation are Ehrlichia spp. and Bacillus odoratum spp. Ehrlichia spp. are pathogenic under normal microbiota conditions and can also cause many diseases, with urinary tract infections being common (12). It has been shown that butyric acid producing bacteria and Streptococcus spp. are reduced and Escherichia spp. are increased in diabetic patients compared with healthy individuals (14). Ehrlichia spp. can be reduced by microbiota transplantation. This is beneficial for patients with T1DM.

After transplantation, changes were found in common between the microbiota of both patients. Both showed an increase in B. cellulosilyticus, which plays an important role in the degradation of cellulose in the human microbiota. Cellulose is a polysaccharide found in plant cell walls and is an important source of dietary fiber. Most bacteria in the human gut are unable to break down plant polysaccharides, and B. cellulosilyticus is the star strain for studying the molecular mechanisms of metabolism of complex plant polysaccharides in fecal microbes (41). Of note, the risk of diabetes is two times higher in people who eat high-fat, low-fiber foods than in people who eat low-fat, high-fiber foods, which shows that dietary fiber has a significant effect on reducing the risk of diabetes (42). The abundance of A. shahil also increased in both patients after transplantation. In patients with compensated and decompensated cirrhosis, fresh fecal metagenome sequences from healthy volunteers and patients with various types of cirrhosis showed a reduction in A. shahil compared to healthy controls (43). In addition, a study conducted in mice with liver cancer showed the potential anti-inflammatory effects of healthy probiotics. Alistipes increased in abundance in the group of mice receiving probiotics. At the species level, A. shahil was shown to be significantly increased in the probiotic group. Some investigators speculate that A. shahil plays a role in tumor suppression like that seen in cancer immunotherapy (44). D. parapsilosis was elevated in both patients after transplantation, the correlation analysis (11) showed that Parabacteroides distasonis is one of the core species in the human microbiota, and its level significantly negatively correlates with disease states such as obesity, NAFLD, and diabetes, suggesting that it may play a positive regulatory role in glucolipid metabolism. This is consistent with our study, in which D. parapsilosis was elevated in both patients with improved glycemic control after transplantation.

We found a significant negative correlation between the genus Faecalibacterium and HOMA-beta. A. muciniphila and Prevotella faecalis have also been shown to be highly abundant human fecal microorganisms in healthy individuals, and reduced levels are associated with inflammation and altered metabolic processes involved in the development of type 2 diabetes (45). Studies have shown that impaired fecal barrier structure and function has been shown to be an important pathogenic process in type 2 diabetes mellitus (T2DM). Dysbiosis of the fecal microbiota is a key factor in the pathogenesis of the diabetic gut. As the most abundant commensal bacterium, Pseudomonas putida plays an important role in fecal homeostasis. One metabolite of P. procumbens has anti-inflammatory potential in inflammatory bowel disease (46). Butyricimonas and Phascolarctobacterium were significantly negatively correlated with Homa-IR. Children with multiple islet autoantibodies (≥−2IA) or T1DM had dysbiosis of the fecal microbiota with lower amounts of Prevotella and Butyricimonas (39). Although this is an interesting finding, studies and trials with more patients are needed to verify the results and to further understand the correlation and potential mechanism for the improvement of insulin resistance by gut microbes.

2HCP was significantly and positively correlated with Streptococcus and Blautia and was significantly and negatively correlated with Phascolarctobacterium. Fasting C-peptide and Streptococcus were significantly positively correlated. A randomized, placebo-controlled trial of T1DM children aged 8 to 17 years treated with placebo or oligofructose-enriched probiotic inulin for 12 weeks revealed a significant increase in C-peptide levels in the group receiving probiotics at 3 months, accompanied by a mild improvement in fecal permeability. The relative abundance of Bifidobacteria in the probiotic group was significantly increased at 3 months but was no longer present after 3 months of washing. At 3 months, the relative abundance of Streptococcus, Roseburia inulinivorans, Terrisporobacter, and Faecalitalea in the placebo group compared to the probiotic group was significantly higher (47). This is in agreement with our study that the 2-hour postprandial C-peptide (2HCP) is significantly and positively correlated with Streptococcus.

One study reported a significant reduction of Blautia in colorectal cancer patients, with potential probiotic properties such as preventing inflammation and promoting SCFA production and other activities to maintain fecal homeostasis (34). Blautia spp. may optimize glycemic control by increasing tolerance to metformin in diabetic patients (48). AMC showed better results in improving homeostatic model assessment of HOMA-IR and plasma triglycerides and had a greater effect on the gut microbiota, and growing evidence suggests that the composition of the gut microbiota is highly correlated with the outcome of T2DM treatment (49).

It has been demonstrated that ferulose oligosaccharides (FOs) and ferulic acid (FA) reduced symptoms in diabetic rats, and that oligofructose reduced the abundance of Lactobacillus, Rumex, Oscillibacter, and Desulfovibrio, while increasing the abundance of Acinetobacter, Phascolarctobacterium, and Turicibacter (50). A. muciniphila and F. prausnitzii are highly abundant human fecal microorganisms in healthy individuals, and their reduced levels are associated with inflammation and altered metabolic processes, which are involved in the development of type 2 diabetes. A. muciniphila and F. prausnitzii are human fecal microorganisms highly abundant in healthy individuals, and their reduced levels are associated with inflammation and alterations in metabolic processes (45).

Metformin can alter the fecal microbiota in type 2 diabetic patients, and gastrofecal tolerance to metformin may be mediated by fecal microbiota. It was found that the early tolerant group had higher abundance of Subdoligranulum, while the intolerant group had higher levels of Subdoligranulum. The tolerant group exhibited an enrichment of Megalomonas and Streptococcus shortum, with lower levels of ruminal cocci in the longitudinal analysis. At the endpoint, the relative abundance of Megamonas, Megamonas rupellensis, and Phascolarctobacterium spp. R. gnavus was higher in the tolerant group than in the non-tolerant group. The numbers of Megamonas, M. rupellensis, Phascolarctobacterium spp., and R. gnavus were higher in the non-tolerant group. PICRUST analysis showed that the activity of the amino acid biosynthesis pathway was lower, and the sugar degradation pathway was higher in the non-tolerant group (48).

It has also been shown that anthocyanin extracts can modulate fecal microbiota in diabetic patients with a decreased abundance of R. torques and L. bacterium 4_1_37FAA and increased oxidative phosphorylation (51).

There has been a dramatic increase in the incidence of the autoimmune disease T1DM. In addition to genetic susceptibility, environmental factors are thought to play a key role in this increase. As with other autoimmune diseases, the gut microbiome is thought to play a potential role in controlling the progression to T1DM in children at a high genetic risk. It has been shown that a high abundance group consisting of two closely related species (B. dorei and B. vulgatus) was significantly higher in the T1DM group than in the control group. Metagenomic sequencing of samples with higher abundance in the B. dorei/Common Bacteroides group prior to seroconversion and longer 16S rRNA sequencing identified this group as B. dorei. The abundance of B. dorei peaked at 7.6 months, 8 months before the appearance of the first islet autoantibodies, suggesting that early changes in the microbiome may help predict T1D autoimmunity in genetically susceptible infants. The reason for the increased abundance of B. dorei in these cases is unclear, but its timing seems to coincide with the introduction of solid foods (52). This is consistent with the results obtained in our study. B. dorei showed a significant positive correlation with FBG.

In conclusion, we found some characteristic bacteria related to clinical indicators of diabetes. More in-depth studies will need to be performed.

## Prospect

Current research on FMT has allowed us to recognize the important role of fecal microbiota in the development of the disease, providing new hope and research basis for the treatment of autoimmune T1DM mellitus by this modality. Many selective and divergent issues still need to be resolved.

### About microbiota donor

Whether the microbiota donor should be selected from healthy allogeneic or autologous donors. autoimmune T1DM mellitus by this modality. Many selective and divergent issues still need to be resolved:

1). Whether the microbiota donor should be selected from healthy allogeneic or autologous donors.
2). Whether the duration of stool storage affects the therapeutic efficacy of microbiota transplantation.
3). Whether the stool should be stored frozen or freeze-dried.
4). Whether patients need to adjust their own fecal microbiota by adjusting their diet, taking probiotics, prebiotics, or antibiotics before the treatment.
5). Which part of the intestine should be selected formicrobiota injection in order to cause less immune rejection and other side effects and better adjust the autoimmune status of the patient. This is necessary because, compared with the small intestine, the large intestine has a larger variety and number of microbes, and the metabolic and immune reactions that occur are more frequent.
6). Whether the length of the treatment period, the number of treatments, and the interval between treatments are closely related to the clinical cure rate of patients.

At present, when patients receive fecal microbiota, the donors are mostly randomized, and the transplantation process is not precise, resulting in low efficiency of microbiota transplantation therapy. Moreover, some microbiota that are not in the demand range of the patient also enter the intestine of the transplanted person, and these microbes may be harmful. The input of harmful bacteria also increases the permeability of the intestine, which requires us to combine the detection of fecal microbiota of patients, analyze the differences in the composition of their fecal microbiota and normal human fecal microbiota, as well as their fecal specific microbiota, beneficial bacteria, harmful bacteria, and other factors.

In addition, there are still many technical problems that need to be solved in FMT. During the process of microbiota transplantation, accidental infections and the input of harmful bacteria will instead reduce the permeability of the fecal mucosa, trigger autoimmune reactions, and accelerate the progress of autoimmune T1DM. It is necessary to improve the development of microbiota injection technology.

## Conclusion

Additionally, although the pre-transplant baseline glycemia was not identical between the two patients, we showed that the number of transplants and the time interval between transplants impacted clinical outcomes; multiple transplants at slightly longer intervals may have an “enhancing” effect in terms of glycemic improvement.

## Data availability statement

The raw sequencing data supporting the conclusions of this article were deposited in the NCBI Sequence Read Archive (SRA) database under bioproject number PRJNA833152 (https://www.ncbi.nlm.nih.gov/sra/PRJNA833152).

## Ethics statement

This study was reviewed and approved by Note: Research ethics number: LHLL2021001, Ethics committee of Longhu People’s Hospital Shantou, China. Written informed consent to participate in this study was provided by the participants’ legal guardian/next of kin. Written informed consent was obtained from the minor(s)’ legal guardian/next of kin for the publication of any potentially identifiable images or data included in this article.

## Author contributions

Conceived and designed the study: KH, FC, LH, and BZ. Participated in investigation: RC, SZ, and LH. Performed formal analysis: BK, DZ, ZW, CX, and BC. Curated the data: BZ, FC, and KH. Wrote the manuscript: FC, RC, SZ, and LH. Supervised the study: KH and BZ. All authors listed have made a substantial, direct, and intellectual contribution to the work and approved it for publication.

## Funding

This work was supported by grants from the Guangdong Science and Technology Special Fund (No.210629086900260), Longhu People’s Hospital, Shantou, China.

## Conflict of interest

Author BC was employed by the Xiamen Treatgut Biotechnology Co., Ltd.

The remaining authors declare that the research was conducted in the absence of any commercial or financial relationships that could be construed as a potential conflict of interest.

The handling editor MZ declared a shared parent affiliation with the authors BZ, CX, and KH at the time of review.

## Publisher’s note

All claims expressed in this article are solely those of the authors and do not necessarily represent those of their affiliated organizations, or those of the publisher, the editors and the reviewers. Any product that may be evaluated in this article, or claim that may be made by its manufacturer, is not guaranteed or endorsed by the publisher.

## Supplementary material

Supplementary Figure 1 PCA samples from patient1 **(A)** and patient 2 **(B)** shown by changes in the microflora at each time visiting point.
