# Supplementary material for: Fecal microbiota transplantation treatment of autoimmune-mediated type 1 diabetes mellitus
Source: Front Immunol. 2022 Aug 12;13:930872. doi: 10.3389/fimmu.2022.930872 (PMC9414079; doi:10.3389/fimmu.2022.930872)
Supplement: Supplementary file 1 [file Table_1.doc]

Your temporary usage period for IBM SPSS Statistics will expire in 5005 days.


GET DATA
  /TYPE=XLSX
  /FILE='/Users/abc/Desktop/Supplementary Table1 .xlsx'
  /SHEET=name 'Sheet1'
  /CELLRANGE=FULL
  /READNAMES=ON
  /DATATYPEMIN PERCENTAGE=95.0
  /HIDDEN IGNORE=YES.
EXECUTE.
DATASET NAME Data Base1 WINDOW=FRONT.
T-TEST GROUPS=Group('D0' 'FMT')
  /MISSING=ANALYSIS
  /VARIABLES=HypersensitiveCRPmgL Bloodroutineleukocyte Bloodsedimentationmmh Procalcitoninngml FBG
    @2HPG HbA1c FCP @2HCP FINS @2INS LactatedehydrogenaseUL PhosphocreatineUL
    PhosphocreatineisoenzymeUL HydroxybutyratedehydrogenaseUL LactatedehydrogenaseUL_A
    AsparticacidtransaminaseUL AlaninetransaminaseUL ASTALT TransglutaminaseUL AlkalinephosphataseUL
    CholinesteraseIUL MonoamineoxidaseUL aLfucosidaseï¼ˆUL TotalbileacidumolL ThetotalproteingL AlbumingL
    GlobulingL UricacidumolL TotalbilirubinumolL DirectbilirubinumolL IndirectbilirubinumolL KmmolL
    NammolL ClmmolL CammolL PmmolL CO2mmolL GlucosemmolL UreanitrogentendencyL CreatinineumolL
    WhiteballratiogL CystatinmgL TotalcholesterolmmolL TriglyceridemmolL HDLCmmolL LDLCmmolL
    ApolipoproteinAgL ApolipoproteinBgL FINSˑFPG22.5 @20FINSFPG3.5
  /CRITERIA=CI(.95).


T-Test


Notes	
Output Created	18-APR-2022 20:16:19	
Comments		
Input	Active Dataset	Data 	
	Filter	<none>	
	Weight	<none>	
	Split File	<none>	
	N of Rows in Working Data File	27	
Missing Value Handling	Definition of Missing	User defined missing values are treated as missing.	
	Cases Used	Statistics for each analysis are based on the cases with no missing or out-of-range data for any variable in the analysis.	
Syntax	T-TEST GROUPS=Group('D0' 'FMT')
  /MISSING=ANALYSIS
  /VARIABLES=HypersensitiveCRPmgL Bloodroutineleukocyte Bloodsedimentationmmh Procalcitoninngml FBG
    @2HPG HbA1c FCP @2HCP FINS @2INS LactatedehydrogenaseUL PhosphocreatineUL
    PhosphocreatineisoenzymeUL HydroxybutyratedehydrogenaseUL LactatedehydrogenaseUL_A
    AsparticacidtransaminaseUL AlaninetransaminaseUL ASTALT TransglutaminaseUL AlkalinephosphataseUL
    CholinesteraseIUL MonoamineoxidaseUL aLfucosidaseï¼ˆUL TotalbileacidumolL ThetotalproteingL AlbumingL
    GlobulingL UricacidumolL TotalbilirubinumolL DirectbilirubinumolL IndirectbilirubinumolL KmmolL
    NammolL ClmmolL CammolL PmmolL CO2mmolL GlucosemmolL UreanitrogentendencyL CreatinineumolL
    WhiteballratiogL CystatinmgL TotalcholesterolmmolL TriglyceridemmolL HDLCmmolL LDLCmmolL
    ApolipoproteinAgL ApolipoproteinBgL FINSˑFPG22.5 @20FINSFPG3.5
  /CRITERIA=CI(.95).	
Resources	Processor Time	00:00:00.03	
	Elapsed Time	00:00:00.00	

[Data Base1] 


Group Statistics	
	Group	N	Mean	Std. Deviation	Std. Error Mean	
Hypersensitive CRP (mg/L)	D0	5	.4060	.72896	.32600	
	FMT	21	1.3671	1.61676	.35281	
Blood routine - leukocyte	D0	5	7.6000	1.04881	.46904	
	FMT	21	5.7476	3.60397	.78645	
Blood sedimentation (mm/h)	D0	5	2.4000	.54772	.24495	
	FMT	21	3.6190	.97346	.21243	
Procalcitonin (ng/ml)	D0	5	1.7080	.93189	.41675	
	FMT	21	.1862	.45648	.09961	
FBG	D0	5	7.0060	1.07568	.48106	
	FMT	21	6.7695	.43553	.09504	
2HPG	D0	5	10.7660	1.35066	.60403	
	FMT	21	7.8190	.65209	.14230	
HbA1c(%)	D0	5	7.5260	.08142	.03641	
	FMT	21	6.8900	.28712	.06266	
FCP	D0	5	.4880	.04324	.01934	
	FMT	21	.3971	.06717	.01466	
2HCP	D0	5	.9080	.07120	.03184	
	FMT	21	.7929	.12834	.02801	
FINS	D0	5	5.7920	.25587	.11443	
	FMT	21	2.4752	.70574	.15400	
2INS	D0	5	11.2240	.33269	.14878	
	FMT	21	6.6152	1.57635	.34399	
Lactate dehydrogenase (U/L)	D0	5	52.7400	19.96216	8.92735	
	FMT	21	36.2333	4.12363	.89985	
Phosphocreatine (U/L)	D0	5	90.8000	6.09918	2.72764	
	FMT	21	79.0476	17.20603	3.75466	
Phosphocreatine isoenzyme (U/L)	D0	5	17.4000	1.14018	.50990	
	FMT	21	15.9524	2.10894	.46021	
Hydroxybutyrate dehydrogenase (U/L)	D0	5	117.4000	23.37306	10.45275	
	FMT	21	120.2857	26.94836	5.88061	
Lactate dehydrogenase (U/L)	D0	5	151.6000	17.30029	7.73692	
	FMT	21	146.5714	5.80148	1.26599	
Aspartic acid transaminase (U/L)	D0	5	23.3800	4.52389	2.02315	
	FMT	21	21.8186	2.30762	.50356	
Alanine transaminase (U/L)	D0	5	19.8120	1.12014	.50094	
	FMT	21	16.6429	1.34036	.29249	
AST/ALT	D0	5	1.1954	.12747	.05701	
	FMT	21	1.3204	.18716	.04084	
Transglutaminase (U/L)	D0	5	10.5980	.27087	.12114	
	FMT	21	12.2600	.85976	.18762	
Alkaline phosphatase (U/L)	D0	5	124.2000	10.37786	4.64112	
	FMT	21	122.0952	11.17544	2.43868	
Cholinesterase (IU/L)	D0	5	7734.0000	1123.31318	502.36093	
	FMT	21	6963.8095	353.50723	77.14160	
Monoamine oxidase (U/L)	D0	5	2.4000	1.14018	.50990	
	FMT	21	3.6190	1.32198	.28848	
a-L-fucosidaseï¼ˆU/L)	D0	5	28.2940	3.58241	1.60210	
	FMT	21	25.7762	1.27863	.27902	
Total bile acid (umol/L)	D0	5	2.7200	.52631	.23537	
	FMT	21	2.2714	.63019	.13752	
The total protein (g/L)	D0	5	76.9040	8.33595	3.72795	
	FMT	21	68.7986	3.16074	.68973	
Albumin (g/L)	D0	5	49.2800	4.79391	2.14390	
	FMT	21	46.1833	1.97346	.43064	
Globulin (g/L)	D0	5	23.6460	4.25237	1.90172	
	FMT	21	23.2448	2.07634	.45309	
Uric acid (umol/L)	D0	5	410.6000	8.14248	3.64143	
	FMT	21	382.5714	20.84843	4.54950	
Total bilirubin (umol/L)	D0	5	11.5560	.53882	.24097	
	FMT	21	12.2962	.83427	.18205	
Direct bilirubin (umol/L)	D0	5	3.5320	.27271	.12196	
	FMT	21	3.4048	.46076	.10055	
Indirect bilirubin (umol/L)	D0	5	7.4560	.13795	.06169	
	FMT	21	8.7576	.70051	.15286	
K(mmol/L)	D0	5	4.2280	.21135	.09452	
	FMT	21	4.1614	.23204	.05064	
Na(mmol/L)	D0	5	137.4000	2.66833	1.19331	
	FMT	21	136.0952	3.54831	.77430	
Cl(mmol/L)	D0	5	100.6800	2.50140	1.11866	
	FMT	21	99.5476	1.88351	.41102	
Ca(mmol/L)	D0	5	2.4180	.10208	.04565	
	FMT	21	2.4129	.09545	.02083	
P(mmol/L)	D0	5	1.3740	.03782	.01691	
	FMT	21	1.3771	.05130	.01119	
CO2(mmol/L)	D0	5	26.5000	1.50333	.67231	
	FMT	21	26.3457	1.76938	.38611	
Glucose(mmol/L)	D0	5	6.9240	1.11314	.49781	
	FMT	21	6.7695	.43553	.09504	
Urea nitrogen (tendency/L)	D0	5	4.9140	.75949	.33966	
	FMT	21	6.0890	.53357	.11643	
Creatinine (umol/L)	D0	5	53.2000	5.89067	2.63439	
	FMT	21	45.9524	5.86068	1.27891	
White ball ratio (g/L)	D0	5	1.9320	.20067	.08974	
	FMT	21	1.9648	.13945	.03043	
Cystatin(mg/L)	D0	5	.5940	.11415	.05105	
	FMT	21	.6305	.04153	.00906	
Total cholesterol (mmol/L)	D0	5	6.3740	.65267	.29188	
	FMT	21	6.2543	.63729	.13907	
Triglyceride (mmol/L)	D0	5	.8060	.06189	.02768	
	FMT	21	1.1043	1.58298	.34543	
HDLC(mmol/L)	D0	5	1.4880	.17796	.07959	
	FMT	21	1.7000	.24698	.05390	
LDLC(mmol/L)	D0	5	3.0780	.46284	.20699	
	FMT	21	3.8648	.66543	.14521	
Apolipoprotein A(g/L)	D0	5	1.6620	.24004	.10735	
	FMT	21	1.4362	.08482	.01851	
Apolipoprotein B(g/L)	D0	5	.8500	.12021	.05376	
	FMT	21	.8986	.10041	.02191	
FINSˑFPG/22.5	D0	5	1.8108	.34913	.15613	
	FMT	21	.7492	.23661	.05163	
20*FINS/(FPG-3.5)	D0	5	35.0260	8.50340	3.80284	
	FMT	21	15.2237	4.11035	.89695	


Independent Samples Test	
	Levene's Test for Equality of Variances	t-test for Equality of Means	
	F	Sig.	t	df	Sig. (2-tailed)	Mean Difference	Std. Error Difference	95% Confidence Interval of the Difference	
								Lower	Upper	
Hypersensitive CRP (mg/L)	Equal variances assumed	.431	.518	-1.283	24	.212	-.96114	.74921	-2.50743	.58514	
	Equal variances not assumed			-2.001	14.797	.064	-.96114	.48036	-1.98623	.06395	
Blood routine - leukocyte	Equal variances assumed	.311	.582	1.122	24	.273	1.85238	1.65094	-1.55498	5.25974	
	Equal variances not assumed			2.023	22.515	.055	1.85238	.91570	-.04415	3.74891	
Blood sedimentation (mm/h)	Equal variances assumed	2.962	.098	-2.673	24	.013	-1.21905	.45598	-2.16015	-.27794	
	Equal variances not assumed			-3.760	11.031	.003	-1.21905	.32423	-1.93243	-.50567	
Procalcitonin (ng/ml)	Equal variances assumed	4.297	.049	5.420	24	.000	1.52181	.28078	.94231	2.10131	
	Equal variances not assumed			3.552	4.467	.020	1.52181	.42849	.37962	2.66400	
FBG	Equal variances assumed	4.048	.056	.802	24	.430	.23648	.29478	-.37191	.84487	
	Equal variances not assumed			.482	4.317	.653	.23648	.49036	-1.08643	1.55938	
2HPG	Equal variances assumed	3.449	.076	7.299	24	.000	2.94695	.40377	2.11361	3.78030	
	Equal variances not assumed			4.749	4.454	.007	2.94695	.62057	1.29102	4.60288	
HbA1c(%)	Equal variances assumed	2.507	.126	4.838	24	.000	.63600	.13147	.36465	.90735	
	Equal variances not assumed			8.776	22.791	.000	.63600	.07247	.48601	.78599	
FCP	Equal variances assumed	1.968	.173	2.862	24	.009	.09086	.03175	.02533	.15639	
	Equal variances not assumed			3.744	9.301	.004	.09086	.02427	.03623	.14548	
2HCP	Equal variances assumed	2.635	.118	1.917	24	.067	.11514	.06007	-.00883	.23912	
	Equal variances not assumed			2.715	11.237	.020	.11514	.04241	.02205	.20824	
FINS	Equal variances assumed	2.367	.137	10.213	24	.000	3.31676	.32477	2.64646	3.98706	
	Equal variances not assumed			17.287	19.089	.000	3.31676	.19186	2.91531	3.71821	
2INS	Equal variances assumed	9.197	.006	6.408	24	.000	4.60876	.71925	3.12430	6.09322	
	Equal variances not assumed			12.297	23.986	.000	4.60876	.37478	3.83522	5.38230	
Lactate dehydrogenase (U/L)	Equal variances assumed	16.173	.000	3.695	24	.001	16.50667	4.46703	7.28717	25.72617	
	Equal variances not assumed			1.840	4.082	.138	16.50667	8.97259	-8.21002	41.22335	
Phosphocreatine (U/L)	Equal variances assumed	.279	.602	1.485	24	.151	11.75238	7.91356	-4.58040	28.08517	
	Equal variances not assumed			2.532	19.510	.020	11.75238	4.64085	2.05613	21.44863	
Phosphocreatine isoenzyme (U/L)	Equal variances assumed	3.040	.094	1.469	24	.155	1.44762	.98560	-.58657	3.48180	
	Equal variances not assumed			2.108	11.628	.057	1.44762	.68687	-.05428	2.94951	
Hydroxybutyrate dehydrogenase (U/L)	Equal variances assumed	.421	.523	-.220	24	.828	-2.88571	13.13010	-29.98490	24.21347	
	Equal variances not assumed			-.241	6.797	.817	-2.88571	11.99340	-31.41856	25.64713	
Lactate dehydrogenase (U/L)	Equal variances assumed	8.742	.007	1.145	24	.264	5.02857	4.39286	-4.03784	14.09498	
	Equal variances not assumed			.641	4.216	.554	5.02857	7.83982	-16.30456	26.36170	
Aspartic acid transaminase (U/L)	Equal variances assumed	2.162	.154	1.120	24	.274	1.56143	1.39408	-1.31580	4.43866	
	Equal variances not assumed			.749	4.508	.491	1.56143	2.08487	-3.97892	7.10177	
Alanine transaminase (U/L)	Equal variances assumed	.079	.782	4.876	24	.000	3.16914	.65000	1.82760	4.51068	
	Equal variances not assumed			5.463	7.029	.001	3.16914	.58008	1.79861	4.53968	
AST/ALT	Equal variances assumed	3.356	.079	-1.406	24	.173	-.12495	.08887	-.30838	.05847	
	Equal variances not assumed			-1.782	8.702	.110	-.12495	.07013	-.28442	.03452	
Transglutaminase (U/L)	Equal variances assumed	3.340	.080	-4.214	24	.000	-1.66200	.39441	-2.47602	-.84798	
	Equal variances not assumed			-7.442	21.483	.000	-1.66200	.22332	-2.12579	-1.19821	
Alkaline phosphatase (U/L)	Equal variances assumed	.158	.694	.383	24	.705	2.10476	5.49689	-9.24026	13.44979	
	Equal variances not assumed			.401	6.416	.701	2.10476	5.24282	-10.52505	14.73457	
Cholinesterase (IU/L)	Equal variances assumed	25.611	.000	2.760	24	.011	770.19048	279.03860	194.28310	1346.09785	
	Equal variances not assumed			1.515	4.190	.201	770.19048	508.24928	-616.00531	2156.38626	
Monoamine oxidase (U/L)	Equal variances assumed	.443	.512	-1.894	24	.070	-1.21905	.64364	-2.54745	.10936	
	Equal variances not assumed			-2.081	6.830	.077	-1.21905	.58585	-2.61136	.17327	
a-L-fucosidaseï¼ˆU/L)	Equal variances assumed	19.433	.000	2.704	24	.012	2.51781	.93113	.59605	4.43957	
	Equal variances not assumed			1.548	4.246	.192	2.51781	1.62622	-1.89615	6.93177	
Total bile acid (umol/L)	Equal variances assumed	.520	.478	1.468	24	.155	.44857	.30558	-.18212	1.07927	
	Equal variances not assumed			1.646	7.033	.144	.44857	.27260	-.19541	1.09256	
The total protein (g/L)	Equal variances assumed	6.346	.019	3.651	24	.001	8.10543	2.22019	3.52318	12.68768	
	Equal variances not assumed			2.138	4.278	.095	8.10543	3.79122	-2.15674	18.36759	
Albumin (g/L)	Equal variances assumed	6.707	.016	2.339	24	.028	3.09667	1.32366	.36476	5.82857	
	Equal variances not assumed			1.416	4.328	.225	3.09667	2.18672	-2.79748	8.99081	
Globulin (g/L)	Equal variances assumed	2.634	.118	.314	24	.756	.40124	1.27901	-2.23852	3.04099	
	Equal variances not assumed			.205	4.464	.846	.40124	1.95495	-4.81109	5.61356	
Uric acid (umol/L)	Equal variances assumed	2.340	.139	2.915	24	.008	28.02857	9.61391	8.18643	47.87072	
	Equal variances not assumed			4.810	17.638	.000	28.02857	5.82735	15.76775	40.28939	
Total bilirubin (umol/L)	Equal variances assumed	1.200	.284	-1.876	24	.073	-.74019	.39446	-1.55432	.07394	
	Equal variances not assumed			-2.451	9.266	.036	-.74019	.30201	-1.42041	-.05997	
Direct bilirubin (umol/L)	Equal variances assumed	2.518	.126	.588	24	.562	.12724	.21651	-.31962	.57409	
	Equal variances not assumed			.805	10.331	.439	.12724	.15806	-.22342	.47790	
Indirect bilirubin (umol/L)	Equal variances assumed	3.702	.066	-4.075	24	.000	-1.30162	.31944	-1.96091	-.64232	
	Equal variances not assumed			-7.896	23.878	.000	-1.30162	.16484	-1.64193	-.96131	
K(mmol/L)	Equal variances assumed	.037	.849	.585	24	.564	.06657	.11382	-.16833	.30147	
	Equal variances not assumed			.621	6.518	.556	.06657	.10723	-.19083	.32398	
Na(mmol/L)	Equal variances assumed	2.036	.166	.767	24	.450	1.30476	1.70055	-2.20500	4.81453	
	Equal variances not assumed			.917	7.801	.387	1.30476	1.42251	-1.99020	4.59973	
Cl(mmol/L)	Equal variances assumed	.905	.351	1.138	24	.266	1.13238	.99513	-.92146	3.18622	
	Equal variances not assumed			.950	5.134	.385	1.13238	1.19178	-1.90726	4.17202	
Ca(mmol/L)	Equal variances assumed	.034	.855	.107	24	.916	.00514	.04806	-.09406	.10434	
	Equal variances not assumed			.102	5.789	.922	.00514	.05018	-.11873	.12902	
P(mmol/L)	Equal variances assumed	.526	.475	-.128	24	.899	-.00314	.02454	-.05378	.04750	
	Equal variances not assumed			-.155	7.967	.881	-.00314	.02028	-.04994	.04366	
CO2(mmol/L)	Equal variances assumed	.000	.986	.179	24	.859	.15429	.85982	-1.62029	1.92886	
	Equal variances not assumed			.199	6.923	.848	.15429	.77529	-1.68313	1.99170	
Glucose(mmol/L)	Equal variances assumed	4.660	.041	.514	24	.612	.15448	.30046	-.46565	.77460	
	Equal variances not assumed			.305	4.296	.775	.15448	.50680	-1.21524	1.52419	
Urea nitrogen (tendency/L)	Equal variances assumed	.940	.342	-4.090	24	.000	-1.17505	.28732	-1.76805	-.58205	
	Equal variances not assumed			-3.273	4.982	.022	-1.17505	.35906	-2.09906	-.25103	
Creatinine (umol/L)	Equal variances assumed	.002	.969	2.483	24	.020	7.24762	2.91885	1.22342	13.27182	
	Equal variances not assumed			2.475	6.040	.048	7.24762	2.92841	.09368	14.40156	
White ball ratio (g/L)	Equal variances assumed	.943	.341	-.435	24	.668	-.03276	.07533	-.18824	.12271	
	Equal variances not assumed			-.346	4.960	.744	-.03276	.09476	-.27696	.21143	
Cystatin(mg/L)	Equal variances assumed	15.219	.001	-1.220	24	.234	-.03648	.02989	-.09817	.02522	
	Equal variances not assumed			-.704	4.255	.518	-.03648	.05185	-.17708	.10413	
Total cholesterol (mmol/L)	Equal variances assumed	.076	.786	.376	24	.710	.11971	.31841	-.53745	.77688	
	Equal variances not assumed			.370	5.961	.724	.11971	.32332	-.67269	.91212	
Triglyceride (mmol/L)	Equal variances assumed	.877	.358	-.415	24	.682	-.29829	.71919	-1.78262	1.18605	
	Equal variances not assumed			-.861	20.253	.399	-.29829	.34654	-1.02058	.42401	
HDLC(mmol/L)	Equal variances assumed	5.637	.026	-1.799	24	.085	-.21200	.11787	-.45528	.03128	
	Equal variances not assumed			-2.206	8.167	.058	-.21200	.09612	-.43287	.00887	
LDLC(mmol/L)	Equal variances assumed	1.127	.299	-2.485	24	.020	-.78676	.31656	-1.44011	-.13341	
	Equal variances not assumed			-3.112	8.495	.013	-.78676	.25284	-1.36396	-.20956	
Apolipoprotein A(g/L)	Equal variances assumed	7.431	.012	3.633	24	.001	.22581	.06215	.09754	.35408	
	Equal variances not assumed			2.073	4.241	.103	.22581	.10893	-.06999	.52161	
Apolipoprotein B(g/L)	Equal variances assumed	.073	.790	-.939	24	.357	-.04857	.05174	-.15536	.05821	
	Equal variances not assumed			-.837	5.410	.438	-.04857	.05805	-.19447	.09732	
FINSˑFPG/22.5	Equal variances assumed	.491	.490	8.244	24	.000	1.06159	.12877	.79581	1.32737	
	Equal variances not assumed			6.455	4.911	.001	1.06159	.16445	.63654	1.48663	
20*FINS/(FPG-3.5)	Equal variances assumed	2.678	.115	7.785	24	.000	19.80234	2.54370	14.55240	25.05227	
	Equal variances not assumed			5.068	4.455	.005	19.80234	3.90718	9.37728	30.22739	
