# Supplementary material for: Fecal microbiota transplantation treatment of autoimmune-mediated type 1 diabetes mellitus
Source: Front Immunol. 2022 Aug 12;13:930872. doi: 10.3389/fimmu.2022.930872 (PMC9414079; doi:10.3389/fimmu.2022.930872)
Supplement: Supplementary file 2 [file Table_2.doc]

Your temporary usage period for IBM SPSS Statistics will expire in 5005 days.


GET DATA
  /TYPE=XLSX
  /FILE='/Users/abc/Desktop/Supplementary Table2.xlsx'
  /SHEET=name 'Sheet1'
  /CELLRANGE=FULL
  /READNAMES=ON
  /DATATYPEMIN PERCENTAGE=95.0
  /HIDDEN IGNORE=YES.
EXECUTE.
DATASET NAME Data Base1 WINDOW=FRONT.
T-TEST GROUPS=Group('D0' 'FMT')
  /MISSING=ANALYSIS
  /VARIABLES=HypersensitiveCRPmgL Bloodroutineleukocyte Bloodsedimentationmmh Procalcitoninngml FBG
    @2HPG HbA1c FCP @2HCP FINS @2INS LactatedehydrogenaseUL PhosphocreatineUL
    PhosphocreatineisoenzymeUL HydroxybutyratedehydrogenaseUL LactatedehydrogenaseUL_A
    AsparticacidtransaminaseUL AlaninetransaminaseUL ASTALT TransglutaminaseUL AlkalinephosphataseUL
    CholinesteraseIUL MonoamineoxidaseUL aLfucosidaseï¼ˆUL TotalbileacidumolL ThetotalproteingL AlbumingL
    GlobulingL UricacidumolL TotalbilirubinumolL DirectbilirubinumolL IndirectbilirubinumolL KmmolL
    NammolL ClmmolL CammolL PmmolL CO2mmolL GlucosemmolL UreanitrogentendencyL CreatinineumolL
    WhiteballratiogL CystatinmgL TotalcholesterolmmolL TriglyceridemmolL HDLCmmolL LDLCmmolL
    ApolipoproteinAgL ApolipoproteinBgL FINSˑFPG22.5 @20FINSFPG3.5
  /CRITERIA=CI(.95).


T-Test


Notes	
Output Created	18-APR-2022 20:28:36	
Comments		
Input	Active Dataset	Data Base1	
	Filter	<none>	
	Weight	<none>	
	Split File	<none>	
	N of Rows in Working Data File	15	
Missing Value Handling	Definition of Missing	User defined missing values are treated as missing.	
	Cases Used	Statistics for each analysis are based on the cases with no missing or out-of-range data for any variable in the analysis.	
Syntax	T-TEST GROUPS=Group('D0' 'FMT')
  /MISSING=ANALYSIS
  /VARIABLES=HypersensitiveCRPmgL Bloodroutineleukocyte Bloodsedimentationmmh Procalcitoninngml FBG
    @2HPG HbA1c FCP @2HCP FINS @2INS LactatedehydrogenaseUL PhosphocreatineUL
    PhosphocreatineisoenzymeUL HydroxybutyratedehydrogenaseUL LactatedehydrogenaseUL_A
    AsparticacidtransaminaseUL AlaninetransaminaseUL ASTALT TransglutaminaseUL AlkalinephosphataseUL
    CholinesteraseIUL MonoamineoxidaseUL aLfucosidaseï¼ˆUL TotalbileacidumolL ThetotalproteingL AlbumingL
    GlobulingL UricacidumolL TotalbilirubinumolL DirectbilirubinumolL IndirectbilirubinumolL KmmolL
    NammolL ClmmolL CammolL PmmolL CO2mmolL GlucosemmolL UreanitrogentendencyL CreatinineumolL
    WhiteballratiogL CystatinmgL TotalcholesterolmmolL TriglyceridemmolL HDLCmmolL LDLCmmolL
    ApolipoproteinAgL ApolipoproteinBgL FINSˑFPG22.5 @20FINSFPG3.5
  /CRITERIA=CI(.95).	
Resources	Processor Time	00:00:00.03	
	Elapsed Time	00:00:00.00	

[Data Base1] 


Group Statistics	
	Group	N	Mean	Std. Deviation	Std. Error Mean	
Hypersensitive CRP (mg/L)	D0	4	.8000	.00000	.00000	
	FMT	10	.8240	.05082	.01607	
Blood routine - leukocyte	D0	4	4.6500	.31091	.15546	
	FMT	10	5.6000	1.72240	.54467	
Blood sedimentation (mm/h)	D0	4	3.2500	1.25831	.62915	
	FMT	10	5.2000	2.04396	.64636	
Procalcitonin (ng/ml)	D0	4	.0500	.00000	.00000	
	FMT	10	.0500	.00000	.00000	
FBG	D0	4	7.4650	.30914	.15457	
	FMT	10	6.0830	1.07906	.34123	
2HPG	D0	4	16.8850	3.96361	1.98181	
	FMT	10	7.8970	1.70547	.53932	
HbA1c(%)	D0	4	6.6250	.03873	.01936	
	FMT	10	6.5450	.07261	.02296	
FCP	D0	4	.5550	.02887	.01443	
	FMT	10	.4280	.13415	.04242	
2HCP	D0	4	1.1525	.04500	.02250	
	FMT	10	.9910	.27473	.08688	
FINS	D0	4	12.3275	.31020	.15510	
	FMT	10	3.3870	1.88404	.59579	
2INS	D0	4	21.6350	.70967	.35484	
	FMT	10	8.2100	2.69848	.85333	
Lactate dehydrogenase (U/L)	D0	4	51.6250	.95699	.47850	
	FMT	10	45.7900	10.29158	3.25448	
Phosphocreatine (U/L)	D0	4	75.5000	3.41565	1.70783	
	FMT	10	72.2000	12.05358	3.81168	
Phosphocreatine isoenzyme (U/L)	D0	4	17.7500	.95743	.47871	
	FMT	10	18.1000	4.53260	1.43333	
Hydroxybutyrate dehydrogenase (U/L)	D0	4	153.7500	5.12348	2.56174	
	FMT	10	132.6000	11.61608	3.67333	
Lactate dehydrogenase (U/L)	D0	4	132.5000	77.69384	38.84692	
	FMT	10	168.0000	16.85889	5.33125	
Aspartic acid transaminase (U/L)	D0	4	20.6875	.41796	.20898	
	FMT	10	19.5570	1.15900	.36651	
Alanine transaminase (U/L)	D0	4	12.0650	.82323	.41161	
	FMT	10	9.8100	.37845	.11968	
AST/ALT	D0	4	1.6975	.13672	.06836	
	FMT	10	1.9978	.16351	.05171	
Transglutaminase (U/L)	D0	4	14.0200	.84427	.42214	
	FMT	10	15.1250	.82890	.26212	
Alkaline phosphatase (U/L)	D0	4	322.2500	6.80074	3.40037	
	FMT	10	369.7000	78.26884	24.75078	
Cholinesterase (IU/L)	D0	4	9324.7500	147.71453	73.85727	
	FMT	10	8376.1000	537.66190	170.02362	
Monoamine oxidase (U/L)	D0	4	2.5000	.57735	.28868	
	FMT	10	2.2000	.42164	.13333	
a-L-fucosidaseï¼ˆU/L)	D0	4	21.5500	.89629	.44814	
	FMT	10	18.7900	1.67362	.52924	
Total bile acid (umol/L)	D0	4	1.3750	.17078	.08539	
	FMT	10	5.0900	1.60031	.50606	
The total protein (g/L)	D0	4	66.0550	1.19358	.59679	
	FMT	10	66.2460	2.74627	.86845	
Albumin (g/L)	D0	4	42.8050	1.28816	.64408	
	FMT	10	44.1910	1.88326	.59554	
Globulin (g/L)	D0	4	23.1675	.37170	.18585	
	FMT	10	21.7560	1.24075	.39236	
Uric acid (umol/L)	D0	4	369.2500	5.43906	2.71953	
	FMT	10	383.2000	60.88386	19.25317	
Total bilirubin (umol/L)	D0	4	15.0875	.20106	.10053	
	FMT	10	22.2110	2.08856	.66046	
Direct bilirubin (umol/L)	D0	4	4.4750	.26300	.13150	
	FMT	10	6.6530	.70577	.22318	
Indirect bilirubin (umol/L)	D0	4	10.0950	.38475	.19238	
	FMT	10	16.5470	1.75221	.55410	
K(mmol/L)	D0	4	4.1500	.26458	.13229	
	FMT	10	4.1490	.28992	.09168	
Na(mmol/L)	D0	4	141.2500	2.87228	1.43614	
	FMT	10	137.3000	3.52042	1.11325	
Cl(mmol/L)	D0	4	101.6500	2.01742	1.00871	
	FMT	10	101.7600	2.63869	.83443	
Ca(mmol/L)	D0	4	2.3525	.07136	.03568	
	FMT	10	2.5360	.09778	.03092	
P(mmol/L)	D0	4	1.3650	.04435	.02217	
	FMT	10	1.4620	.14673	.04640	
CO2(mmol/L)	D0	4	22.0000	.81650	.40825	
	FMT	10	23.8700	.97303	.30770	
Glucose(mmol/L)	D0	4	192.5300	370.31334	185.15667	
	FMT	10	6.0830	1.07906	.34123	
Urea nitrogen (tendency/L)	D0	4	4.6700	.17068	.08534	
	FMT	10	5.5580	.80080	.25324	
Creatinine (umol/L)	D0	4	51.7500	2.21736	1.10868	
	FMT	10	55.5000	2.95334	.93393	
White ball ratio (g/L)	D0	4	1.8200	.02944	.01472	
	FMT	10	2.0150	.03866	.01222	
Cystatin(mg/L)	D0	4	.5300	.03651	.01826	
	FMT	10	.6820	.07300	.02308	
Total cholesterol (mmol/L)	D0	4	3.4250	.17078	.08539	
	FMT	10	4.6320	.68558	.21680	
Triglyceride (mmol/L)	D0	4	.5250	.03697	.01848	
	FMT	10	.5140	.09477	.02997	
HDLC(mmol/L)	D0	4	1.4775	.02500	.01250	
	FMT	10	1.4990	.22571	.07137	
LDLC(mmol/L)	D0	4	1.8650	.08185	.04093	
	FMT	10	2.8500	.44811	.14170	
Apolipoprotein A(g/L)	D0	4	1.2325	.03862	.01931	
	FMT	10	3.2930	6.57385	2.07883	
Apolipoprotein B(g/L)	D0	4	.6150	.02887	.01443	
	FMT	10	7.5190	21.25094	6.72014	
FINSˑFPG/22.5	D0	4	4.0900	.19673	.09837	
	FMT	10	.9694	.26113	.08257	
20*FINS/(FPG-3.5)	D0	4	62.4477	4.76803	2.38401	
	FMT	10	44.0150	64.24318	20.31548	


Independent Samples Test	
	Levene's Test for Equality of Variances	t-test for Equality of Means	
	F	Sig.	t	df	Sig. (2-tailed)	Mean Difference	Std. Error Difference	95% Confidence Interval of the Difference	
								Lower	Upper	
Hypersensitive CRP (mg/L)	Equal variances assumed	5.952	.031	-.922	12	.375	-.02400	.02604	-.08073	.03273	
	Equal variances not assumed			-1.494	9.000	.170	-.02400	.01607	-.06035	.01235	
Blood routine - leukocyte	Equal variances assumed	1.227	.290	-1.071	12	.305	-.95000	.88725	-2.88315	.98315	
	Equal variances not assumed			-1.677	10.321	.123	-.95000	.56642	-2.20677	.30677	
Blood sedimentation (mm/h)	Equal variances assumed	.722	.412	-1.755	12	.105	-1.95000	1.11140	-4.37153	.47153	
	Equal variances not assumed			-2.162	9.243	.058	-1.95000	.90200	-3.98234	.08234	
Procalcitonin (ng/ml)	Equal variances assumed	45.714	.000	2.613	12	.023	.00000	.00000	.00000	.00000	
	Equal variances not assumed			3.016	7.789	.017	.00000	.00000	.00000	.00000	
FBG	Equal variances assumed	2.239	.160	2.466	12	.030	1.38200	.56037	.16106	2.60294	
	Equal variances not assumed			3.689	11.606	.003	1.38200	.37461	.56272	2.20128	
2HPG	Equal variances assumed	6.486	.026	6.147	12	.000	8.98800	1.46224	5.80204	12.17396	
	Equal variances not assumed			4.376	3.454	.016	8.98800	2.05388	2.91219	15.06381	
HbA1c(%)	Equal variances assumed	.134	.720	2.055	12	.062	.08000	.03893	-.00481	.16481	
	Equal variances not assumed			2.663	10.468	.023	.08000	.03004	.01348	.14652	
FCP	Equal variances assumed	4.649	.052	1.834	12	.092	.12700	.06926	-.02390	.27790	
	Equal variances not assumed			2.834	10.771	.017	.12700	.04481	.02812	.22588	
2HCP	Equal variances assumed	4.911	.047	1.142	12	.276	.16150	.14139	-.14655	.46955	
	Equal variances not assumed			1.800	10.111	.102	.16150	.08974	-.03816	.36116	
FINS	Equal variances assumed	1.485	.246	9.220	12	.000	8.94050	.96963	6.82785	11.05315	
	Equal variances not assumed			14.522	10.122	.000	8.94050	.61564	7.57100	10.31000	
2INS	Equal variances assumed	1.992	.184	9.600	12	.000	13.42500	1.39840	10.37814	16.47186	
	Equal variances not assumed			14.527	11.362	.000	13.42500	.92417	11.39881	15.45119	
Lactate dehydrogenase (U/L)	Equal variances assumed	2.198	.164	1.105	12	.291	5.83500	5.28046	-5.67013	17.34013	
	Equal variances not assumed			1.774	9.380	.108	5.83500	3.28947	-1.56058	13.23058	
Phosphocreatine (U/L)	Equal variances assumed	2.931	.113	.527	12	.608	3.30000	6.25773	-10.33442	16.93442	
	Equal variances not assumed			.790	11.577	.445	3.30000	4.17679	-5.83749	12.43749	
Phosphocreatine isoenzyme (U/L)	Equal variances assumed	3.012	.108	-.150	12	.884	-.35000	2.33947	-5.44727	4.74727	
	Equal variances not assumed			-.232	10.720	.821	-.35000	1.51116	-3.68669	2.98669	
Hydroxybutyrate dehydrogenase (U/L)	Equal variances assumed	.778	.395	3.444	12	.005	21.15000	6.14141	7.76902	34.53098	
	Equal variances not assumed			4.723	11.630	.001	21.15000	4.47837	11.35794	30.94206	
Lactate dehydrogenase (U/L)	Equal variances assumed	13.015	.004	-1.446	12	.174	-35.50000	24.55173	-88.99363	17.99363	
	Equal variances not assumed			-.905	3.114	.430	-35.50000	39.21104	-157.74837	86.74837	
Aspartic acid transaminase (U/L)	Equal variances assumed	2.887	.115	1.864	12	.087	1.13050	.60655	-.19105	2.45205	
	Equal variances not assumed			2.680	11.999	.020	1.13050	.42190	.21124	2.04976	
Alanine transaminase (U/L)	Equal variances assumed	3.326	.093	7.244	12	.000	2.25500	.31128	1.57678	2.93322	
	Equal variances not assumed			5.261	3.520	.009	2.25500	.42866	.99808	3.51192	
AST/ALT	Equal variances assumed	1.159	.303	-3.228	12	.007	-.30027	.09303	-.50295	-.09758	
	Equal variances not assumed			-3.503	6.685	.011	-.30027	.08571	-.50489	-.09564	
Transglutaminase (U/L)	Equal variances assumed	.006	.940	-2.243	12	.045	-1.10500	.49267	-2.17844	-.03156	
	Equal variances not assumed			-2.224	5.487	.072	-1.10500	.49690	-2.34893	.13893	
Alkaline phosphatase (U/L)	Equal variances assumed	8.902	.011	-1.182	12	.260	-47.45000	40.15127	-134.93211	40.03211	
	Equal variances not assumed			-1.899	9.333	.089	-47.45000	24.98327	-103.66020	8.76020	
Cholinesterase (IU/L)	Equal variances assumed	1.910	.192	3.401	12	.005	948.65000	278.91360	340.94946	1556.35054	
	Equal variances not assumed			5.118	11.490	.000	948.65000	185.37240	542.75966	1354.54034	
Monoamine oxidase (U/L)	Equal variances assumed	1.929	.190	1.089	12	.297	.30000	.27538	-.30000	.90000	
	Equal variances not assumed			.943	4.351	.395	.30000	.31798	-.55549	1.15549	
a-L-fucosidaseï¼ˆU/L)	Equal variances assumed	1.809	.204	3.075	12	.010	2.76000	.89753	.80446	4.71554	
	Equal variances not assumed			3.980	10.437	.002	2.76000	.69349	1.22352	4.29648	
Total bile acid (umol/L)	Equal variances assumed	1.933	.190	-4.522	12	.001	-3.71500	.82147	-5.50483	-1.92517	
	Equal variances not assumed			-7.239	9.497	.000	-3.71500	.51322	-4.86679	-2.56321	
The total protein (g/L)	Equal variances assumed	2.545	.137	-.132	12	.897	-.19100	1.45066	-3.35173	2.96973	
	Equal variances not assumed			-.181	11.688	.859	-.19100	1.05373	-2.49371	2.11171	
Albumin (g/L)	Equal variances assumed	.782	.394	-1.336	12	.206	-1.38600	1.03740	-3.64629	.87429	
	Equal variances not assumed			-1.580	8.300	.151	-1.38600	.87722	-3.39620	.62420	
Globulin (g/L)	Equal variances assumed	4.875	.047	2.188	12	.049	1.41150	.64513	.00588	2.81712	
	Equal variances not assumed			3.251	11.721	.007	1.41150	.43415	.46307	2.35993	
Uric acid (umol/L)	Equal variances assumed	11.598	.005	-.447	12	.663	-13.95000	31.23516	-82.00557	54.10557	
	Equal variances not assumed			-.717	9.352	.491	-13.95000	19.44429	-57.68526	29.78526	
Total bilirubin (umol/L)	Equal variances assumed	3.335	.093	-6.647	12	.000	-7.12350	1.07172	-9.45858	-4.78842	
	Equal variances not assumed			-10.663	9.407	.000	-7.12350	.66807	-8.62487	-5.62213	
Direct bilirubin (umol/L)	Equal variances assumed	2.844	.117	-5.888	12	.000	-2.17800	.36987	-2.98389	-1.37211	
	Equal variances not assumed			-8.408	11.996	.000	-2.17800	.25904	-2.74242	-1.61358	
Indirect bilirubin (umol/L)	Equal variances assumed	6.475	.026	-7.130	12	.000	-6.45200	.90493	-8.42367	-4.48033	
	Equal variances not assumed			-11.000	10.828	.000	-6.45200	.58654	-7.74547	-5.15853	
K(mmol/L)	Equal variances assumed	.225	.644	.006	12	.995	.00100	.16790	-.36482	.36682	
	Equal variances not assumed			.006	6.105	.995	.00100	.16095	-.39121	.39321	
Na(mmol/L)	Equal variances assumed	.473	.505	1.981	12	.071	3.95000	1.99377	-.39406	8.29406	
	Equal variances not assumed			2.174	6.863	.067	3.95000	1.81709	-.36425	8.26425	
Cl(mmol/L)	Equal variances assumed	1.894	.194	-.074	12	.942	-.11000	1.47778	-3.32980	3.10980	
	Equal variances not assumed			-.084	7.361	.935	-.11000	1.30911	-3.17500	2.95500	
Ca(mmol/L)	Equal variances assumed	1.376	.264	-3.376	12	.006	-.18350	.05436	-.30194	-.06506	
	Equal variances not assumed			-3.887	7.743	.005	-.18350	.04721	-.29300	-.07400	
P(mmol/L)	Equal variances assumed	1.407	.258	-1.271	12	.228	-.09700	.07631	-.26327	.06927	
	Equal variances not assumed			-1.886	11.743	.084	-.09700	.05143	-.20932	.01532	
CO2(mmol/L)	Equal variances assumed	.305	.591	-3.376	12	.006	-1.87000	.55395	-3.07696	-.66304	
	Equal variances not assumed			-3.658	6.660	.009	-1.87000	.51122	-3.09146	-.64854	
Glucose(mmol/L)	Equal variances assumed	25.571	.000	1.702	12	.114	186.44700	109.54156	-52.22355	425.11755	
	Equal variances not assumed			1.007	3.000	.388	186.44700	185.15698	-402.80289	775.69689	
Urea nitrogen (tendency/L)	Equal variances assumed	11.468	.005	-2.148	12	.053	-.88800	.41338	-1.78869	.01269	
	Equal variances not assumed			-3.323	10.745	.007	-.88800	.26723	-1.47788	-.29812	
Creatinine (umol/L)	Equal variances assumed	.619	.447	-2.274	12	.042	-3.75000	1.64918	-7.34325	-.15675	
	Equal variances not assumed			-2.587	7.508	.034	-3.75000	1.44962	-7.13133	-.36867	
White ball ratio (g/L)	Equal variances assumed	.005	.945	-9.013	12	.000	-.19500	.02164	-.24214	-.14786	
	Equal variances not assumed			-10.191	7.393	.000	-.19500	.01913	-.23976	-.15024	
Cystatin(mg/L)	Equal variances assumed	.736	.408	-3.905	12	.002	-.15200	.03893	-.23682	-.06718	
	Equal variances not assumed			-5.165	10.940	.000	-.15200	.02943	-.21682	-.08718	
Total cholesterol (mmol/L)	Equal variances assumed	13.611	.003	-3.401	12	.005	-1.20700	.35487	-1.98019	-.43381	
	Equal variances not assumed			-5.180	11.200	.000	-1.20700	.23301	-1.71873	-.69527	
Triglyceride (mmol/L)	Equal variances assumed	1.265	.283	.221	12	.829	.01100	.04977	-.09745	.11945	
	Equal variances not assumed			.312	11.958	.760	.01100	.03521	-.06575	.08775	
HDLC(mmol/L)	Equal variances assumed	5.484	.037	-.186	12	.856	-.02150	.11588	-.27397	.23097	
	Equal variances not assumed			-.297	9.534	.773	-.02150	.07246	-.18403	.14103	
LDLC(mmol/L)	Equal variances assumed	14.096	.003	-4.267	12	.001	-.98500	.23086	-1.48800	-.48200	
	Equal variances not assumed			-6.678	10.348	.000	-.98500	.14750	-1.31215	-.65785	
Apolipoprotein A(g/L)	Equal variances assumed	1.900	.193	-.612	12	.552	-2.06050	3.36811	-9.39899	5.27799	
	Equal variances not assumed			-.991	9.002	.348	-2.06050	2.07892	-6.76323	2.64223	
Apolipoprotein B(g/L)	Equal variances assumed	1.922	.191	-.634	12	.538	-6.90400	10.88787	-30.62663	16.81863	
	Equal variances not assumed			-1.027	9.000	.331	-6.90400	6.72015	-22.10602	8.29802	
FINSˑFPG/22.5	Equal variances assumed	.260	.619	21.389	12	.000	3.12062	.14590	2.80274	3.43850	
	Equal variances not assumed			24.298	7.480	.000	3.12062	.12843	2.82084	3.42040	
20*FINS/(FPG-3.5)	Equal variances assumed	1.885	.195	.559	12	.586	18.43272	32.94504	-53.34836	90.21379	
	Equal variances not assumed			.901	9.244	.390	18.43272	20.45488	-27.65368	64.51911	
