# Supplementary figures and images for: Fecal microbiota transplantation treatment of autoimmune-mediated type 1 diabetes mellitus
Source: Front Immunol. 2022 Aug 12;13:930872. doi: 10.3389/fimmu.2022.930872 (PMC9414079; doi:10.3389/fimmu.2022.930872)

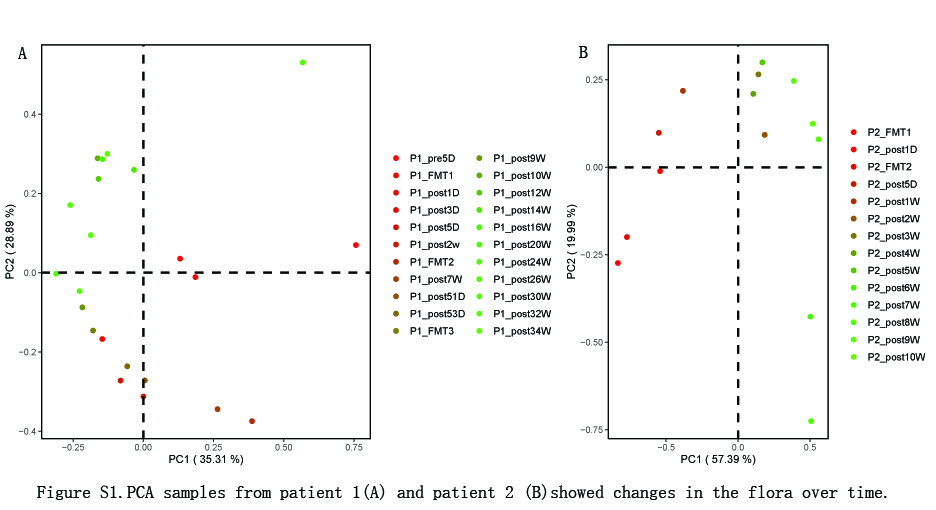

Supplement: Supplementary Figure 1 — PCA samples from patient1 (A) and patient 2 (B) shown by changes in the microflora at each time visiting point. [file Image_1.tif]
